# Immobilization of transition metal (Ni, Cu, and Co)-based colloidal nanoscale materials for the oxygen evolution reaction

**DOI:** 10.1039/d6ra07054j

**Published:** 2026-10-14

**Authors:** Sajid Ullah, Syeda Tabeer Zahra, Khizer Hayat Khan, R. Nawaz, Norah Alomayrah, M. S. Al-Buriahi, Aiyeshah Alhodaib, Zeinebou Yahya, Ziaur Rehman, Akhtar Munir

**Affiliations:** a Department of Chemistry, Quaid-i-Azam University Islamabad 45320 Pakistan akhtarmunir@qau.edu.pk zrehman@qau.edu.pk; b Center for Applied Mathematics and Bioinformatics (CAMB), Gulf University for Science and Technology 32093 Hawally Kuwait; c Department of Physics, College of Science, Princess Nourah bint Abdulrahman University P. O. Box 84428 Riyadh 11671 Saudi Arabia; d Department of Physics, Sakarya University Sakarya Turkey; e Department of Physics, College of Science, Qassim University Buraydah 51452 Al-Qassim Saudi Arabia

## Abstract

The rational design of high-performance electrocatalysts for the oxygen evolution reaction (OER) is the holy grail for clean energy conversion and storage, aimed at mitigating the ever-growing energy demands. However, the inherent sluggish kinetics of the OER remain the primary bottleneck, consequently impeding overall water splitting for efficient hydrogen production. In this regard, ultrasmall metal nanoclusters (NCs, size ≤ 3 nm) or nanoscale materials with tunable physicochemical properties have recently emerged, offering unparalleled opportunities for manipulating the catalytic efficiency and electronic structure at the atomic level. Herein, we report a rational design of transition metal (Ni, Cu, and Co)-based colloidal nanoscale materials immobilized on an N-doped carbon (NC) support for the OER. The structure, size, and electronic properties were investigated using UV-vis, photoluminescence (PL), Fourier transform infrared (FTIR), and energy-dispersive X-ray (EDX) spectroscopies, high-resolution transmission electron microscopy (HR-TEM), and X-ray photoelectron spectroscopy (XPS). Electrochemical measurements demonstrated encouraging OER performance, where NiNCs@NC, CoNCs@NC, and CuNCs@NC achieved a current density of 10 mA cm^−2^ at operational voltages of 1.45, 1.48, and 1.51 V, respectively. Reasonable Tafel slopes and electrochemically active surface area (ECSA) were observed, following the trend of operational potential, indicating an inherent link between the structure–property relationship and kinetics of the OER. Long-term electrochemical testing revealed good durability over 10 hours for all samples, with NiNCs@NC presenting exceptional persistent performance for over 50 hours. Comprehensive structural and electrochemical studies indicated that the improved OER performance stems from the synergistic integrations of thiol-protected monometallic nanoscale materials with the NC support. In a nutshell, this study provides a viable strategy for immobilizing surface-functionalized colloidal nanoscale materials for electrocatalysis, efficiently bridging homogeneous and heterogeneous catalysis.

## Abstract 2

The rational design of high-performance electrocatalysts for the oxygen evolution reaction (OER) is the holy grail for clean energy conversion and storage, aimed at mitigating the ever-growing energy demands.

## Graphical abstract

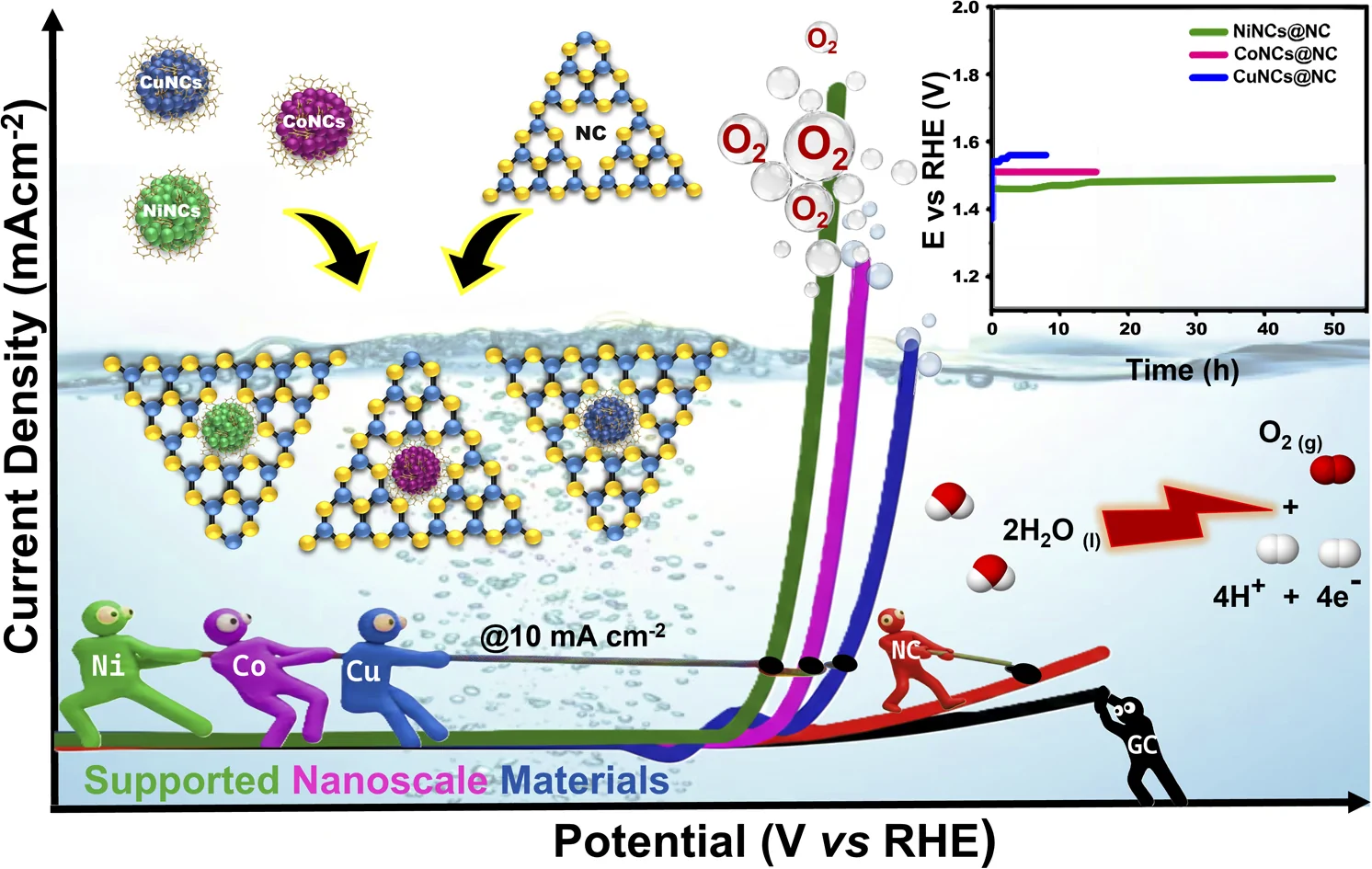

## 1. Introduction

Fossil fuels are currently the leading source of energy around the globe; however, they produce an immense amount of CO_2_, ultimately resulting in various environmental issues.^1,2^ Hence, significant attention has been devoted by the scientific community to the exploration of green and sustainable energy conversion schemes.^3,4^ Being a neat and clean energy carrier with the highest mass energy density (120–140 MJ kg^−1^), molecular hydrogen (H_2_) is a potential candidate for a safe and sustainable future with generous environmental benignity. Currently, one of the most widely accepted methods for H_2_ production is steam reforming, but this process diminishes the environmental merits of hydrogen energy due to the release of CO and CO_2_ as byproducts.^3^ Consequently, electrochemical water splitting (WS) has been extensively explored as a cleaner and sustainable alternative to produce molecular H_2_ and O_2_ without any harmful impacts.^5^ However, behind this elegant simplicity lies a cascade of atomic-scale events that determine the efficiency, cost, and scalability. More specifically, the OER, which is the anodic half-cell reaction of water electrolysis, is widely accepted as the primary bottleneck due to its complex four-electron and four-proton transfer mechanism involving multiple reaction intermediates (OH*, O*, and OOH*).^2,6,7^ This sluggish reaction requires a high overpotential, resulting in significant energy losses and reducing the overall efficiency of water splitting. Although noble metal oxides, like IrO_2_ and RuO_2_, exhibit excellent OER activity, their high cost, scarcity and limited long-term stability hinder their large-scale commercialization.^8,9^

Consequently, the rational design and development of highly efficient, low-cost electrode materials are crucial to accelerate the reaction kinetics to enable an efficient WS process near the thermodynamic limit.^10^ Earth-abundant transition metal-based electrocatalysts can be a cost-effective and efficient choice to substitute Pt- and RuO_2_-/IrO_2_-based catalytic systems due to their rich redox chemistry and widespread availability. However, these materials have slow intrinsic kinetics and minimal active surface exposure.^11,12^ To overcome these limitations, various structural engineering strategies have been developed.^13–15^ Among them, the structural downscaling to metal nanoparticles (NPs) has been widely explored for manipulating the dynamics of the active surface area and size-dependent electronic effects.^16^ Despite these benefits, NPs under harsh operating conditions tend to undergo agglomeration, incomplete surface utilization, and surface reconstruction, necessitating further structural optimization for advanced catalysis.^17,18^

Unlike their bulk counterparts, downscaling of materials to the nanoscale level is one of the flourishing approaches to explore their exciting size-dependent catalytic properties.^19^ Metal nanoclusters (NCs) with a size of ≤3 nm are emerging nanoscale materials having unique size-dependent physicochemical properties with a small number of core atoms. These materials have a high redox nature that can fluctuate, and their intrinsic catalytic behavior can be selectively tuned by controlling their size and core atoms at the atomic level, making them catalytically accessible. Their properties can be further enhanced using suitable surrounding ligands and underlying supports.^20^

Recent progress in ligand-protected nanoclusters has demonstrated the importance of atomic-level structural modulation in nanocatalysis. Kwon *et al.* reported cobalt oxide nanoclusters with abundant unsaturated Co sites and Co–O bonds, which enhanced Co 3d and O 2p hybridization and activated the lattice oxygen mechanism (LOM) to subsequently decrease the overpotential and enhance the stability.^21^ Similarly, atomically precise Cu_14_/Cu_13_ and Ni_4_/Ni_6_ nanoclusters have been developed, which demonstrated that atomic-level control over the composition and surface linkers of the clusters markedly improves their electrocatalytic activities.^20,22^ The influence of ligand engineering on the OER activity was also investigated for noble metal Au_25_(SR)_18_ nanoclusters, where modulation of the surface electronic structure and charge transfer ability resulted in significantly enhanced electrocatalytic performance.^23^ Atomic precision and ligand engineering have substantially improved the intrinsic activity of metal nanoclusters. However, the presence of organic linkers coated on the core of the clusters and their small size increase their solubility in different solvents, and thus, they need to be immobilized on a suitable support for catalysis. Recent efforts have shifted more towards their integration with conductive supports to harness strong metal–support electronic coupling, accelerate charge transfer, and achieve superior catalytic performance. Recently, Seong *et al.* anchored atomically precise Ni_6_ nanoclusters onto nickel foam *via* a dip-coating approach and achieved a remarkably low overpotential.^24^ Similarly, Yu *et al.* further developed nitrogen-doped Ni nanoclusters confined within an amorphous carbon matrix, where the synergistic effects of special confinement and nitrogen doping enhanced the electrical conductivity and stabilized the ultrasmall clusters with a low overpotential output.^25^ The importance of the conductive support was also explored by Zhao *et al.*, where Au_25_ nanoclusters anchored on CoSe nanosheets substantially promoted interfacial electron transfer, leading to accelerated OER kinetics.^26^ Furthermore, a series of 3d transition metal-based nanoclusters immobilized on NiO nanosheets were reported by Marimuthu *et al.*, demonstrating that strong metal–support interactions effectively regulated the adsorption energies of key reaction intermediates, leading to enhanced OER and hydrogen evolution reaction (HER) kinetics.^12^ Based on these intriguing catalytic and dynamic properties, NCs are emerging as nanoscale materials in catalysis science. However, metal NCs inherently suffer from higher surface energy, leading to aggregation, structural instability, surface oxidation, and leaching, which collectively diminish their catalytic properties.^27–30^

In our previous work, a thiol-functionalized strategy was developed to synthesize ultrasmall colloidal nanoclusters (Ni, Co, and Cu) for OER.^31^ Here, in this study, the pre-synthesized colloidal NiNCs, CoNCs, and CuNCs were immobilized on NC nanosheets *via* simple sonication. Exfoliation of these NCs generated thin nanosheets with abundant accessible nitrogen sites, enabling effective surface optimization while facilitating the uniform anchoring of NCs on the support. The intimate interfacial coupling arising from the strong interactions between NCs and the nitrogen-rich matrix can induce substantial electronic modulation, promote rapid charge transfer kinetics, and maximize the exposure of the catalytically active sites. The present work demonstrates a simple strategy for developing supported metal NCs electrocatalysts, providing a scientifically sound catalytic platform with enhanced activity, durability, and reaction kinetics towards the OER.

## 2. Experimental

### 2.1. Materials and methods

Nickel(ii) acetylacetonate (Ni(acac)_2_, ≥99%, Sigma-Aldrich), cobalt chloride (CoCl_2_, ≥99%, Sigma-Aldrich), copper chloride (CuCl_2_, ≥99%, Sigma-Aldrich), potassium hydroxide (KOH, ≥85%, Sigma-Aldrich), melamine (99%, Macklin), sodium borohydride (NaBH_4_, ≥96%, Sigma-Aldrich), glutathione (GSH, ≥98%, TCI), tetraoctylammonium bromide (TOAB, ≥98%, Sigma-Aldrich) and 2-phenylethanethiol (≥99%, Sigma-Aldrich) were used in this study. Different solvents like tetrahydrofuran (THF), toluene, dichloromethane (DCM), methanol, ethanol, and deionized (DI) water were used after distillation.

### 2.2. Synthesis of the thiolated Cu and Co nanoclusters

The modified Brust–Schiffrin and ligand exchange methods are the two versatile methods used for the synthesis of noble metal (Au, Pd, and Pt) nanoclusters.^32^ Recently, we optimized a synthetic strategy for transition metal (Cu and Co) NCs using the above-mentioned method (Brust–Schiffrin) by tuning the stoichiometric ratio of the metal, ligands, and surfactants to control the size of the metals in the sub-nanometric regime. For the synthesis of CoNCs, an optimized stoichiometric amount of 30 mg of CoCl_2_ (0.23 mmol) and 90 mg of TOAB (0.16 mmol) were dissolved in 15 mL of distilled THF at room temperature. After complete dissolution, PET (0.1 mL, 0.76 mmol) was added to the reaction mixture, resulting in the appearance of a brown color denoting the possible complexation process. The reaction mixture was stirred for 2 hours, followed by the addition of an ice-cold aqueous solution of NaBH_4_ (2.11 mmol, 80 mg in 2 mL DI water). The solution turned dark brown and was further stirred for the next 24 hours to complete the aging process. A similar procedure was followed for the synthesis of CuNCs, except using 50 mg of CuCl_2_ (0.37 mmol) as the metal precursor and 75 mL of toluene as the solvent, along with TOAB (400 mg, 0.73 mmol), PET (400 µL, 3.1 mmol), and NaBH_4_ (3.7 mmol, 140 mg in 10 mL DI water).

### 2.3. Synthesis of the thiolated Ni nanoclusters

For the synthesis of NiNCs, the size-focusing methodology was adopted, as reported previously, where glutathione-stabilized large nanoparticles (NPs) were first synthesized by a simple reduction method.^20^ Briefly, Ni(acac)_2_ (96 mg, 0.374 mmol) and GSH (461 mg, 1.5 mmol) were dissolved in 20 mL of acetone and stirred for 20 min at room temperature. The reaction mixture was cooled to 0 °C followed by the rapid addition of freshly prepared aqueous NaBH_4_ (142 mg, 3.75 mmol) under vigorous stirring. The glutathione-stabilized NPs were treated in a mixture of toluene/methanol (10 : 1) with PET (140 µL, 1.07 mmol) at 80 °C for 4 hours. Later, the toluene phase with solubilized clusters was separated and concentrated by evaporating the solvent. NCs were then precipitated by the addition of methanol, and the same process was repeated three times to remove the unreacted ligand, surfactant, and physically adsorbed organic linker. The final pure material was dispersed in DCM for further use.^33^

### 2.4. Synthesis of graphitic N-doped carbon (NC)

The NC support was synthesized by annealing melamine in static air using a muffle furnace: first heated to 500 °C (2.5 °C min^−1^ ramp rate, 3 h hold) and then to 550 °C (1 h hold) to complete the polymerization, yielding NC as a yellow powder.

### 2.5. Synthesis of the supported monometallic nanoscale materials on NC

The synthesized colloidal NCs were individually loaded onto the NC *via* a simple exfoliation and dispersion method. Initially, 200 mg of NC was finely ground and ultrasonically dispersed in methanol. The previously synthesized NCs (∼150 µL) were added to the dispersion and then stirred at room temperature for 12 h. The temperature was slowly increased for the controlled evaporation of the solvent (70%) to ensure the saturation and loading of NCs. The remaining solution was centrifuged, and the clear supernatant was removed. The centrifugation process was repeated several times to remove the physically adsorbed NCs, and the samples were dried in vacuum at 60 °C for further analysis (Scheme 1).

**Scheme 1:**
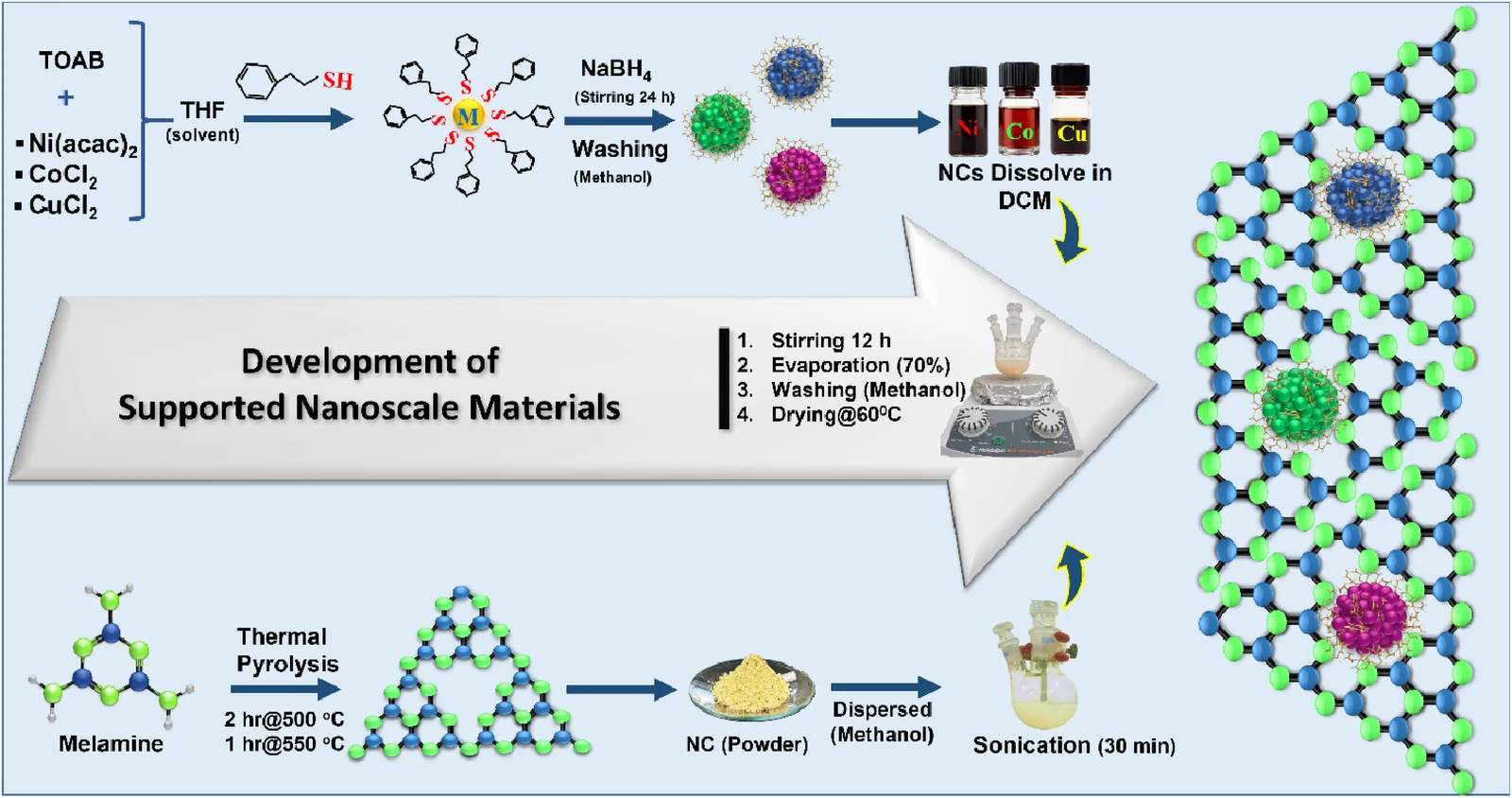
Synthesis of the nanoclusters supported on N-doped carbon (NCs@NC). TOAB: tetraoctylammonium bromide and acac: acetylacetonate.

### 2.6. Characterization

The optical properties and electronic transitions of the synthesized materials were examined by UV-vis spectroscopy using a Shimadzu UV-1800 UV-vis spectrophotometer over the wavelength range of 250–800 nm. PL spectra were recorded using a PerkinElmer LS 55 luminescence spectrometer to investigate the emission characteristics of the synthesized materials. The excitation wavelength was selected based on the maximum absorption wavelength obtained from the UV-vis spectra, while the emission spectra were collected over the wavelength range of 300–800 nm. FTIR spectra were recorded using a Thermo Scientific Nicolet 6700 FTIR spectrophotometer in the range of 4000–400 cm^−1^. The elemental composition of all the NCs@NC samples was determined using the INCAx-Act EDX system. HR-TEM (JEM-2100F, JEOL, operated at 200 kV) was performed to investigate the size and uniform distribution of NCs over the NC. XPS analysis was carried out using a PHI 5000 VersaProbe II spectrometer to examine the elemental composition, oxidation states, chemical environment, and purity of the synthesized materials (NCs@NC).

### 2.7. Electrochemical measurements

The electrocatalytic OER performance was evaluated using a Gamry Reference 600 potentiostat in a conventional three-electrode electrochemical cell. A modified glassy carbon (GC) electrode with a surface area of 0.07 cm^2^ was deployed as the working catalyst electrode; Ag/AgCl and a platinum wire were used as the reference and counter electrodes, respectively. All electrochemical measurements were carried out in a 1 M KOH aqueous electrolyte at room temperature. Cyclic voltammetry (CV) was performed over a potential range encompassing the OER region (0.0–0.8 V *vs.* Ag/AgCl, equivalent to ∼1.02–1.82 V *vs.* RHE) to evaluate the electrocatalytic performance. Electrochemical impedance spectroscopy (EIS) measurements were carried out over a frequency range of 1 Hz to 1 MHz at a potential close to the OER onset potential to study the interfacial charge-transfer kinetics and resistance of the electrocatalysts. The working electrodes were fabricated using a simple drop-casting method and subsequently dried under ambient conditions.

## 3. Results and discussion

### 3.1. Colloidal nanoscale materials (NCs)

The Brust–Shiffrin and ligand exchange methods are cornerstone synthetic approaches in nanochemistry for the preparation of thiolated metal NCs with precise control over their size, composition, and surface functionality.^34^ The synthesis of NCs is generally challenging due to uncontrolled nucleation, rapid aggregation, and surface instability. These approaches overcome the aforementioned limitations using a two-phase liquid–liquid system in which metal ions are transferred from the aqueous phase to the organic phase *via* a phase transfer agent like TOAB, enabling homogeneous dissolution and controlled reduction in an organic medium with NaBH_4_ in the presence of a thiol-based ligand. Thiols strongly coordinate to the metal surface, forming a protective ligand shell that stabilizes the nascent clusters and prevents their aggregation, thereby allowing precise control over particle growth at the nanoscale level.^35^ This bottom-up synthetic method is particularly significant in terms of simplicity and reproducibility, making it a widely adopted approach for the synthesis of ultrasmall metal nanoclusters.

NiNCs, CoNCs, and CuNCs were synthesized using our previously reported method.^31^ The organic ligand, *i.e.*, 2-phenylethanethiol (PET), consists of an aromatic ring linked to sulfur through two carbon atoms that exerts a mild electronic impact through inductive and resonance effects. Therefore, it is likely that the donor capacity of sulfur is increased toward the metals (Ni, Co and Cu) *via* the S–M bond, thereby modulating the electronic environment to efficiently stabilize the core atoms in metal NCs.^36,37^ Hence, the inherent structural ability to show strong interactions with metals and stabilize the metallic particles in an organic medium is worth considering not only to maximize the colloidal stability but also to increase the shelf life. The metal-to-ligand ratios were experimentally optimized in accordance with the metal precursors to achieve NC formation and their colloidal stability. The colloidal stability at the nanoscale level is highly linked to size and surface chemistry, which may vary from metal to metal. This variation in the ligand amount reflects the difference in precursor reduction behavior, metal thiolate intermediate formation, nucleation tendency, and surface stabilization requirements.^38^ Therefore, the experimentally optimized PET concentration employed for NiNCs, CoNCs, and CuNCs synthesis ensures efficient growth regulation, suppression of agglomeration, and formation of well-dispersed ultrasmall metal NCs.

### 3.2. Immobilization on the support

Colloidal nanoscale materials behave like mini molecules with abundant accessible sites for catalysis.^35^ Promisingly, their structural stability and catalytic potential can be further improved by uniformly loading them onto a suitable conducting support like the NC. Such a judicious approach potentially advocates the synergistic interactions being responsible for the robustness through interfacial charge transfer and possible optimization of the adsorption/desorption energies of the intermediates.^39–41^ N-doped carbon is a nitrogen-rich framework composed of heptazine/triazine units, offering abundant coordination sites for anchoring metal NCs.

The tri-*s*-triazine units of the NC comprise different types of nitrogen, such as pyrrolic, pyridinic, and graphitic nitrogen. The graphitic nitrogen, embedded within the sp^2^-conjugated framework, acts as an electron donor, thus modulating the electronic structure of the support and enhancing the charge transfer between the support and active metal sites. Additionally, the pyridinic nitrogen possesses a localized lone pair of electrons that imparts Lewis basicity and can serve as a strong coordination site for metal NCs.^42^ Meanwhile, pyrrolic nitrogen contributes to the delocalized π system and generates defect-rich environments that can facilitate the adsorption and activation of intermediates. Therefore, the presence of graphitic, pyridinic, and pyrrolic nitrogen modulates the electronic properties, metal-anchoring capabilities, and catalytic activity of the catalyst.^43,44^ However, the metal NCs possess a dual electronic and structural environment: (1) delocalized metal–metal bonding within the core and (2) metal–ligand coordination at the surface, forming the ligand–metal–ligand motif.^45^ In a hybrid system, the direct interaction between thiolated sulfur and the nitrogen of NC is generally unfavorable because both are electron-rich centers. In contrast, the strong M–S bond in NCs and M–N coordination with the NC can work together to provide anchoring and effective electronic transfer, resulting in a hybrid structure with reasonable electronic contact.^46^ Nitrogen acts as an electron donor, promoting M–N bond formation and enabling d–p orbital hybridization between metal d-orbitals and nitrogen p-orbitals.^47^ This interaction could induce charge redistribution across the interface, and the optimum electronic structure is responsible for the significant enhancement in the OER performance.^48^

### 3.3. Structural characterization of the supported nanoscale materials (NCs@NC)

The structural, optical, and surface properties of the synthesized NCs@NC samples were systematically examined using advanced complementary characterization techniques. Understanding the effect of support immobilization on the electronic structure of NCs@NC is essential for correlating their optical and catalytic properties. Therefore, UV-vis absorption spectroscopy was employed to systematically study the metal-core electronic transitions of NCs before and after their immobilization, revealing the influence of support interactions on their optical behaviors. The appearance of multiple absorption peaks in the 300–550 nm region and absence of a surface plasmon resonance (SPR) band in all spectra provided a clear clue of NC formation (Fig. 1a). These multiple absorption peaks reflect the electronic transitions within the metal core that usually occur in metal MCs in the size range of 1–2 nm.^49^ The NC spectra (Fig. 1b) show a characteristic peak at 320 nm that can be attributed to the sp^2^-conjugated C–N (π–π* transition). The supported materials (NiNCs@NC, CoNCs@NC, and CuNCs@NC) exhibit a broad absorption profile and red-shifted absorption edge, widely attributed to the charge-transfer interactions between NCs and the NC framework (Fig. 1b). The UV-vis absorption spectra of metal NCs immobilized on the NC support revealed significant changes in optical properties, indicating the interactions between NCs and the support that may alter the electronic structure of the host material.^50^ The corresponding Tauc plot analysis (Fig. 1c) of the UV-vis absorption spectra revealed a progressive narrowing of the optical band gap upon NC loading from 2.74 eV for pristine NC to 2.62, 2.10, and 2.06 eV for CuNCs@NC, CoNCs@NC, and NiNCs@NC, respectively. Band gap narrowing is commonly associated with the formation of new electronic states near the valence and conduction edges, which can reasonably facilitate interfacial charge transfer. Similar mechanisms have been proposed for Pt-, Pd- and Au-modified NCs, where the metal-induced electronic states significantly alter the optical and electronic properties of the support.^51^ Furthermore, the broadness in the spectral intensity of peaks may arise due to the coupling of NCs immobilized on the NC, which shows the coordination of the metal core to the hetero-site (C and N) of the NC that facilitates the two-way interfacial charge transfer among the ligand, metal and support.^52,53^ Further, PL spectroscopy was employed to probe the charge-carrier recombination dynamics of pristine NC and NCs@NC samples (Fig. 1d). All samples exhibited a broad emission band centered at ∼465 nm, characteristic of the band edge emission of the NC, arising from the radiative recombination of photogenerated electron–hole pairs. Notably, the emission maxima remain almost unchanged after NC loading, reflecting that the electronic framework of the NC is retained. The PL intensity decreases significantly in the order of NC > CoNCs@NC > CuNCs@NC > NiNCs@NC, revealing a progressive suppression of electron–hole recombination. This trend suggests more efficient interfacial charge transfer between the NC and NCs, where NiNCs@NC displayed the strongest charge transfer, followed by CuNCs@NC and then CoNCs@NC. To further elucidate the charge-transfer behavior, excitation wavelength-dependent PL spectra were recorded for all the synthesized materials, confirming the reproducibility of the emission characteristics over a wide excitation wavelength range, as shown in Fig. S1a–d. The observed fluorescence quenching shows that the supported NCs act as electron acceptors, promoting charge separation by facilitating electron transfer between the NC and NCs.^54^

**Fig. 1:**
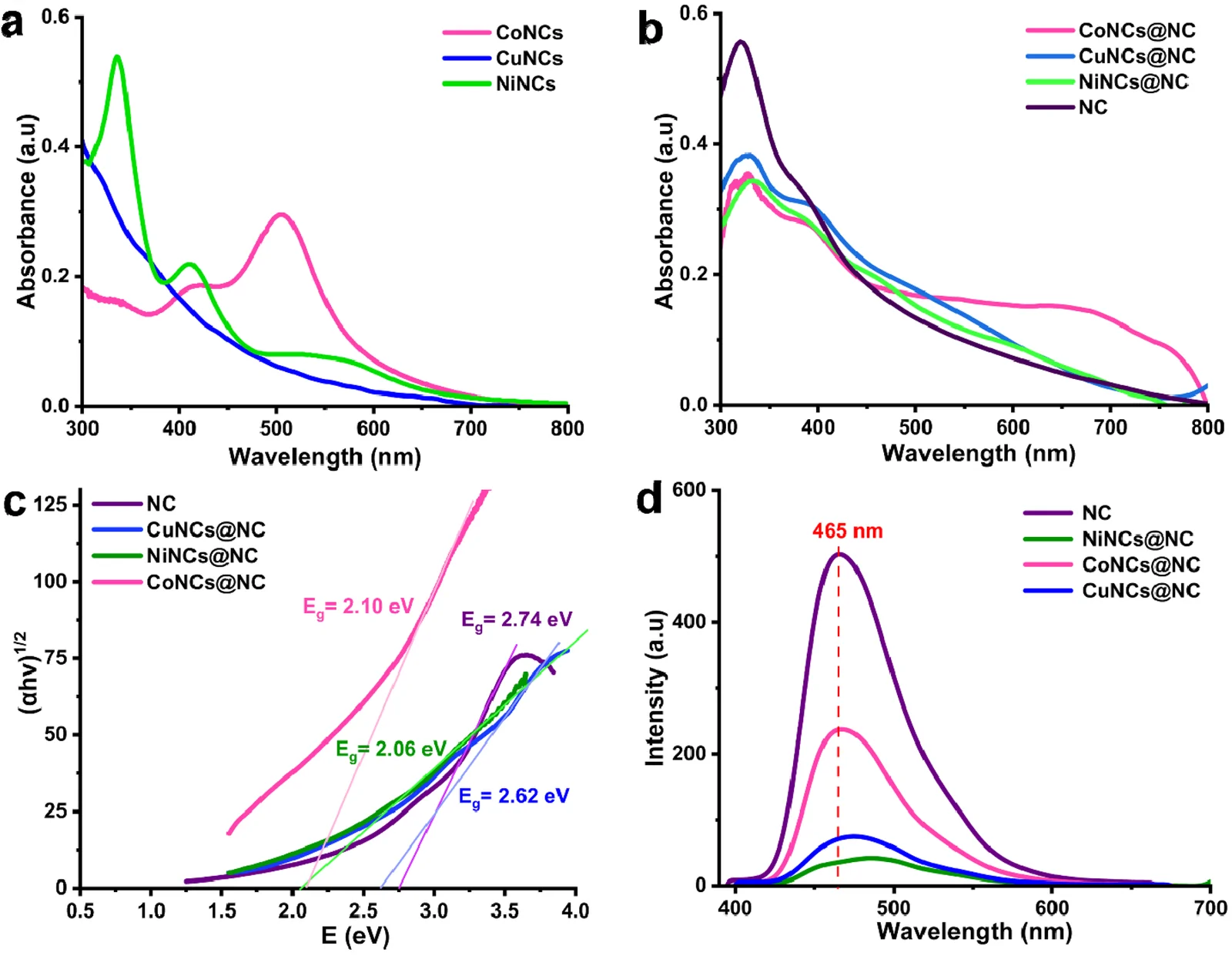
(a) UV-vis absorption spectra of the ligand-protected NiNCs, CoNCs and CuNCs. (b) UV-vis absorption spectra of NC, NiNCs@NC, CoNCs@NC and CuNCs@NC. (c) Tauc plots derived from the UV-vis absorption spectra for estimating the optical band gaps of NC, NiNCs@NC, CoNCs@NC and CuNCs@NC. (d) PL emission spectra of NC, NiNCs@NC, CoNCs@NC and CuNCs@NC.

To further probe the surface functionality and investigate the coordination environment, FTIR spectroscopy was performed for all the synthesized materials (Fig. 2). Commercial PET exhibits a characteristic S–H stretching band near 2500–2600 cm^−1^. Hence, the absence of this band across all the NCs@NC samples suggests that the thiol moiety (S–H) is no longer present in its free protonated form, indicating metal–thiolate bond formation. The characteristic heptazine (tri-*s*-triazine) ring breathing mode, a fingerprint vibration of pristine NC, appears at 800 cm^−1^. Upon immobilization of NCs, no significant difference was observed in this band, which appeared at 805 cm^−1^ for NiNCs@NC, 803 cm^−1^ for CoNCs@NC, and 802 cm^−1^ for CuNCs@NC, indicating the preservation of the NC framework.^55,56^ The peak appearing at around 3160 cm^−1^ in all FTIR spectra corresponds to N–H stretching, suggesting the partial presence of terminal NH_2_ residues (mainly at the edges of the 2D sheets).^57^ Collectively, these FTIR results support the successful immobilization of PET-protected NCs on the support. The preservation of the metal–thiolate coordination, together with the characteristic vibrational features of the NC framework, suggests that both NCs and the support retain their structural integrity after loading.

**Fig. 2:**
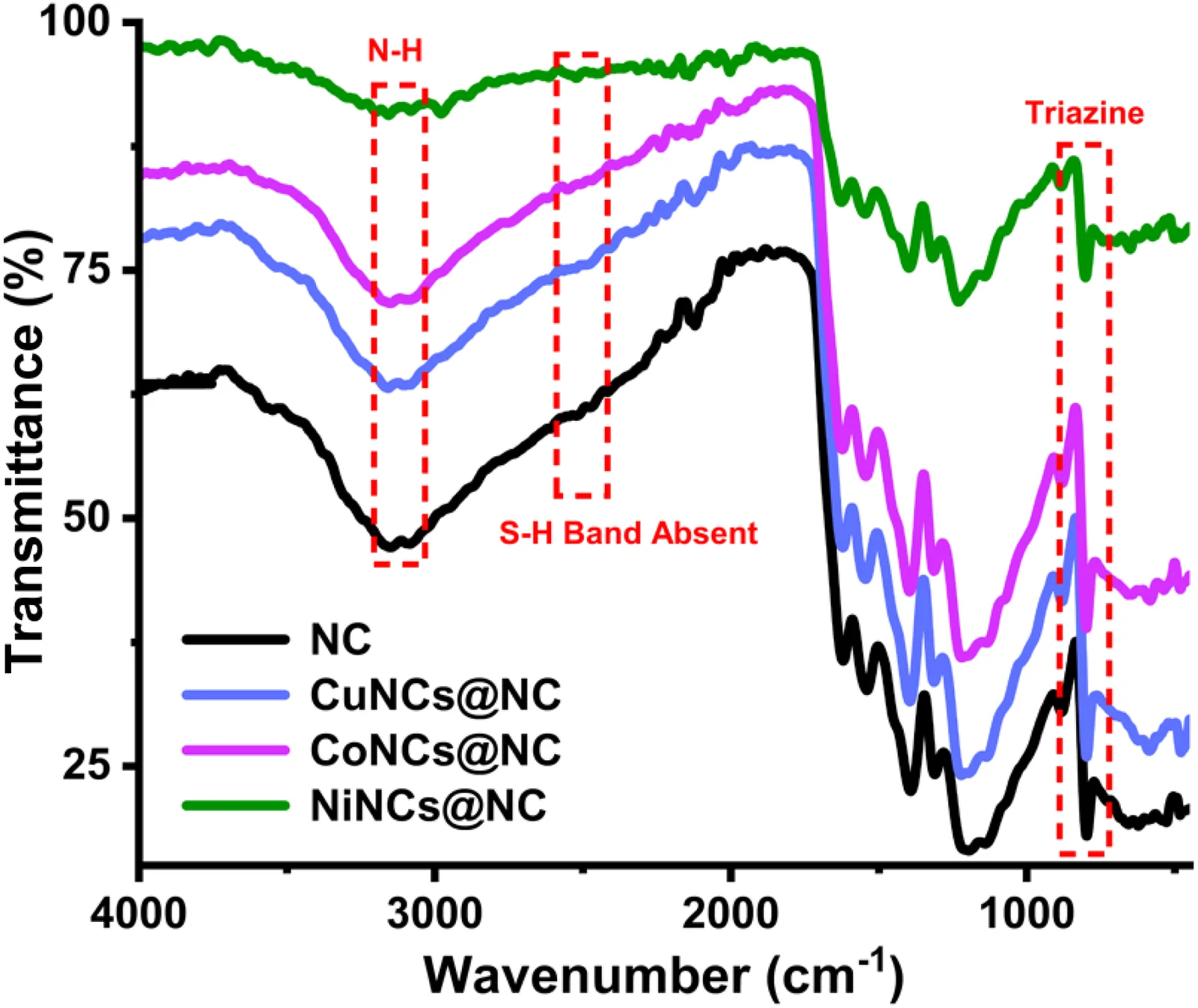
FTIR spectra of NC, NiNCs@NC, CoNCs@NC and CuNCs@NC.

Elemental analysis by EDX verified the compositional integrity of the NC through its characteristic carbon–nitrogen composition (Fig. 3b). The NC was further characterized using XRD, where the peak at around 27° indicates the graphitic carbon, which is expected after thermal treatment at high temperatures (Fig. 3a). The SEM images indicate the bulk nature (low magnification) and layered form of the 2D nature (high magnification) (Fig. 3c and d, respectively). The microstructural morphology was further investigated using TEM after exfoliation (Fig. 3e and f), which confirms the layered sheet-like smooth morphology originating from the stacking of the internal layers. Promisingly, the layered structure can be exfoliated by the ultrasonication process to improve the conductivity and trapping ability of the exfoliated layers. Therefore, we used the NC as the underlying support for the loading of NiNCs, CoNCs, and CuNCs.

**Fig. 3:**
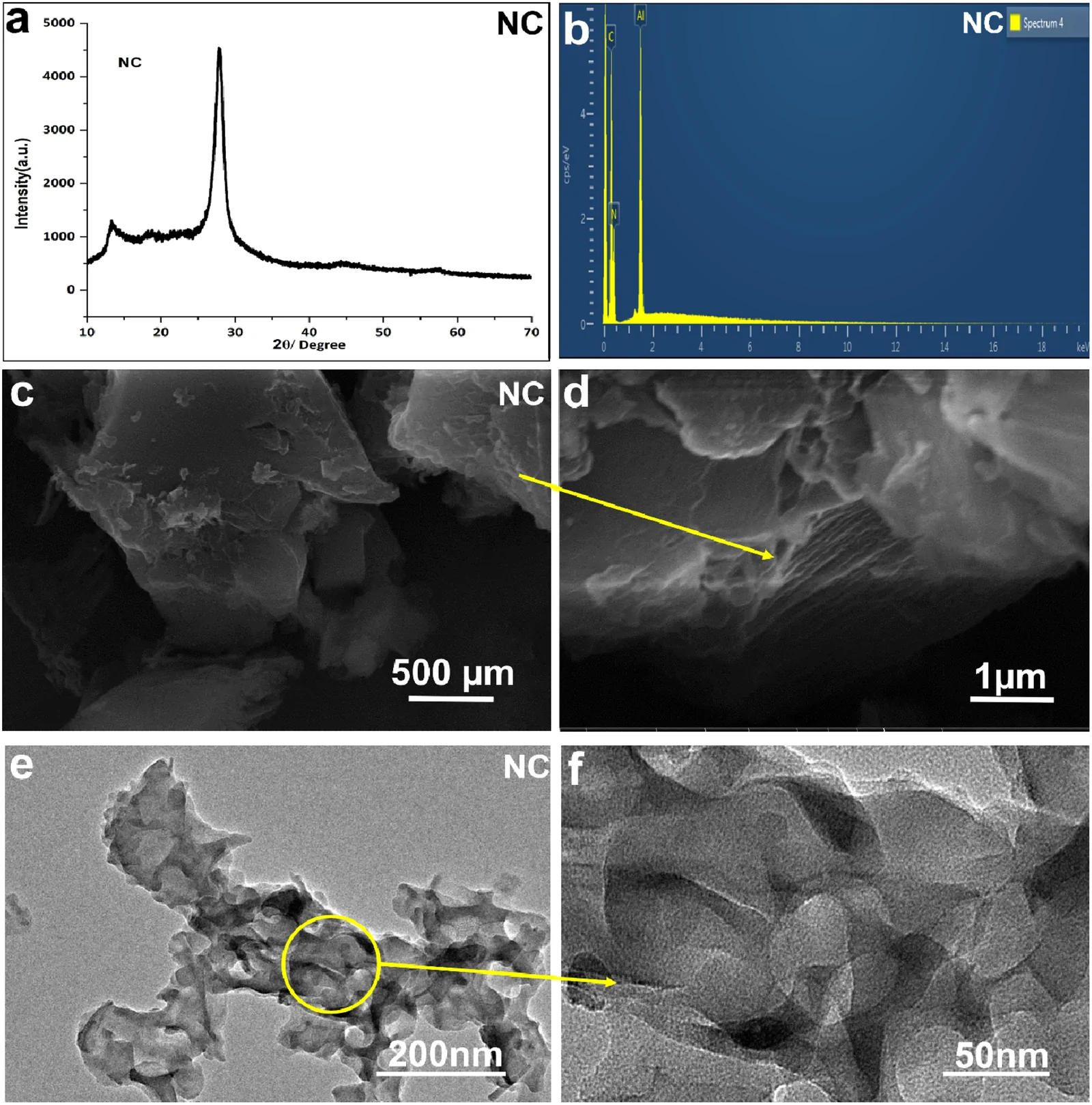
(a) XRD pattern of the nitrogen-doped carbon (NC). (b) EDX analysis of the NC. (c and d) SEM images of the NC and (e and f) TEM images of the NC under low and high magnifications, respectively.

The elemental composition of the synthesized NCs@NC was confirmed using EDX in order to verify the presence of all the expected elements (C, S, and N) including metals like Ni, Co and Cu in the respective samples. Nitrogen is not shown in the Co and Ni EDX spectra (Fig. S2-4a); this may be due to the poor sensitivity of EDX towards elements of low atomic weight. Moreover, due to the ultrasmall size and limited crystallinity of NCs in the sub-nanometric regime, scanning electron microscopy (SEM) and conventional powdered X-ray diffraction (XRD) are not reliable to resolve their morphology and crystal structure.^31,58^ Therefore, the distribution of NCs on the support was systematically investigated by HR-TEM and compared with the bare support. NCs exhibit a relatively uniform size distribution, with no pronounced aggregations observed across the analyzed region. However, transition metals generally exhibit poor contrast against carbonaceous support, making their direct visualization challenging. Nevertheless, the repeated HR-TEM analysis of different regions of NCs@NC samples in comparison with that of the bare support revealed noticeable small dots and changes in surface contrast after NC immobilization. The HR-TEM micrographs of NiNCs@NC, CoNCs@NC and CuNCs@NC taken at different magnifications are illustrated in Fig. 4a–f and S2-4(b–e). The comparative and contrast analysis with the bare NC indicates that the light contrast represents the thin layers of NC, while the dark spots represent the small metal NCs, which may arise from the higher electron density of metal NCs relative to the NC support. The HR-TEM micrographs of NiNCs@NC (Fig. 4a and b) show a well-dispersed, ultrasmall, and uniform granular microstructure with discrete assemblies over the support, reflecting that these tiny aggregates grow along the edges of the thin layers of NC despite their agglomeration. The surface assembly on the support in a particular pattern indicates that the atoms along the edge are less coordinated and may preferably be available for the immobilization of NCs. Similarly, the HR-TEM images of Co and Cu show the uniform distribution of NCs with a contrasting feature against the bare NC, as shown in Fig. 4c–f. It is noteworthy that the contrast and boundaries of the tiny aggregates in the case of Cu are much better, probably accounting for the higher atomic weight or favorable aging time during the reaction. Owing to the ultrasmall dimensions of NCs and possible overlap of their contrast with that of the carbon support, well-resolved lattice fringes are not consistently marked for NiNCs@NC and CoNCs@NC. This limitation is expected for NCs, where the small number of atoms and cluster orientation can strongly influence the visibility of lattice features in the HR-TEM analysis. In comparison, clearer lattice fringes were observed for CuNCs@NC, providing the possible local evidence of structural ordering at the nanoscale level. The observed lattice features are, therefore, dependent not only on the intrinsic structure of NCs but also on their orientation and the local contrast conditions under the electron beam. Accordingly, the lattice fringes observed for CuNCs@NC (Fig. 4f (inset) and S5e) may be considered as representative of the locally resolved structural features rather than evidence of uniformly developed lattice fringes throughout NCs. Overall, the distribution of NCs throughout the substrate without considerable aggregation is clearly shown by the HR-TEM images, indicating the efficacy of the immobilization strategy, which is also reflected in catalysis.

**Fig. 4:**
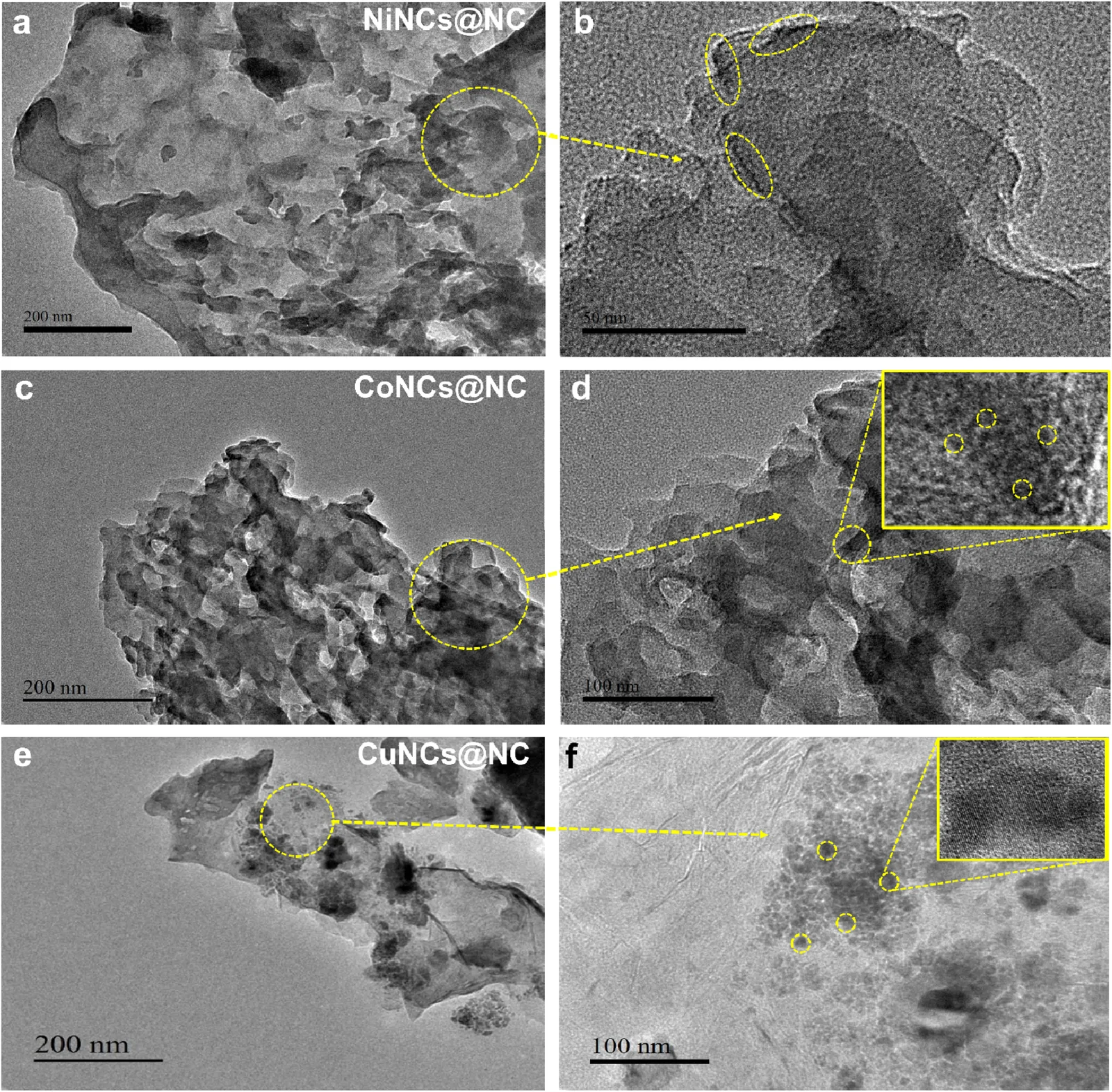
HR-TEM images of (a and b) NiNCs@NC, (c and d) CoNCs@NC and (e and f) CuNCs@NC.

X-ray photoelectron spectroscopy (XPS) was used to examine the elemental composition, oxidation state, and chemical environment of all the NCs@NC. The survey spectra of thiol-stabilized NiNCs, CoNCs, and CuNCs loaded on the NC indicate the presence of expected elements like N, C, and O, and the respective metals (Fig. 5a). The core level spectra of C 1s, N 1s, S 2p, Ni 2p, and O 1s are shown in Fig. 5b–f, respectively. The multiple peaks in the C 1s spectrum (Fig. 5b) may arise due to the presence of C–C, C=C/C–S, and C–N=C, indicating the presence of an organic linker and N-doped carbon network.^59^ The N 1s spectrum confirms the formation of the nitrogen-doped carbonaceous support by exhibiting three primary peaks attributed to pyridinic, pyrrolic, and graphitic nitrogen (Fig. 5c). The peaks that appear at binding energies of ∼397.5 eV and ∼400 eV correspond to the nitrogen atom located at the edge or defect site of the carbon network, where it is bonded to two carbon atoms in the pyridine and pyrrole rings, respectively. The graphitic nitrogen peak appears at ∼402 eV and corresponds to the quaternary nitrogen attached to three carbon atoms in the carbon network.^60^ The well-defined nitrogen environment retention after NC loading is consistent with the support-mediated stabilization of NCs. The high-resolution S 2p spectrum, as shown in Fig. 5d, was deconvoluted and corresponds to distinct sulfur environments. The S 2p_3/2_ peaks centered at approximately 162.2 eV and 163.4 eV can be assigned to the sulfur coordinated with Ni through metal–thiolate (Ni–S), respectively, confirming the preservation of the metal–thiolate coordination after immobilization. A weak peak at a lower binding energy of ∼161.9 eV may be assigned to a small amount of terminal or weakly coordinated sulfur species such as free thiolate (S^−^) or residual thiol (–SH). Meanwhile, the higher binding energy doublet located at ∼168–171 eV is unequivocally assigned to the oxidized sulfur species (S–O/S=O) because of exposure to air or oxidation under a high-energy beam. Further, the core level spectrum of Ni 2p shows that there is a metal present in two distinct oxidation states (Ni^0/2+^).^20^ The peak towards the lower binding energy (∼852.56 eV) is due to the metallic state, while the peaks appearing at higher binding energies, *i.e.*, ∼854.31 eV and ∼861.58 eV, can be assigned to Ni^2+^ 2p_3/2_ and Ni^2+^ 2p_1/2_, respectively (Fig. 5e).^59^ Based on the synthetic conditions, we generally expect a low oxidation state of metal (preferably the metallic state); however, various factors like high surface energy of small particles, exposure to air, oxidation of free sulfur, possible adsorption of water molecules, and etching during analysis may partially oxidize the metal surface.^61^ Consequently, the presence and possible effect of oxygen can be seen in all XPS spectra.

**Fig. 5:**
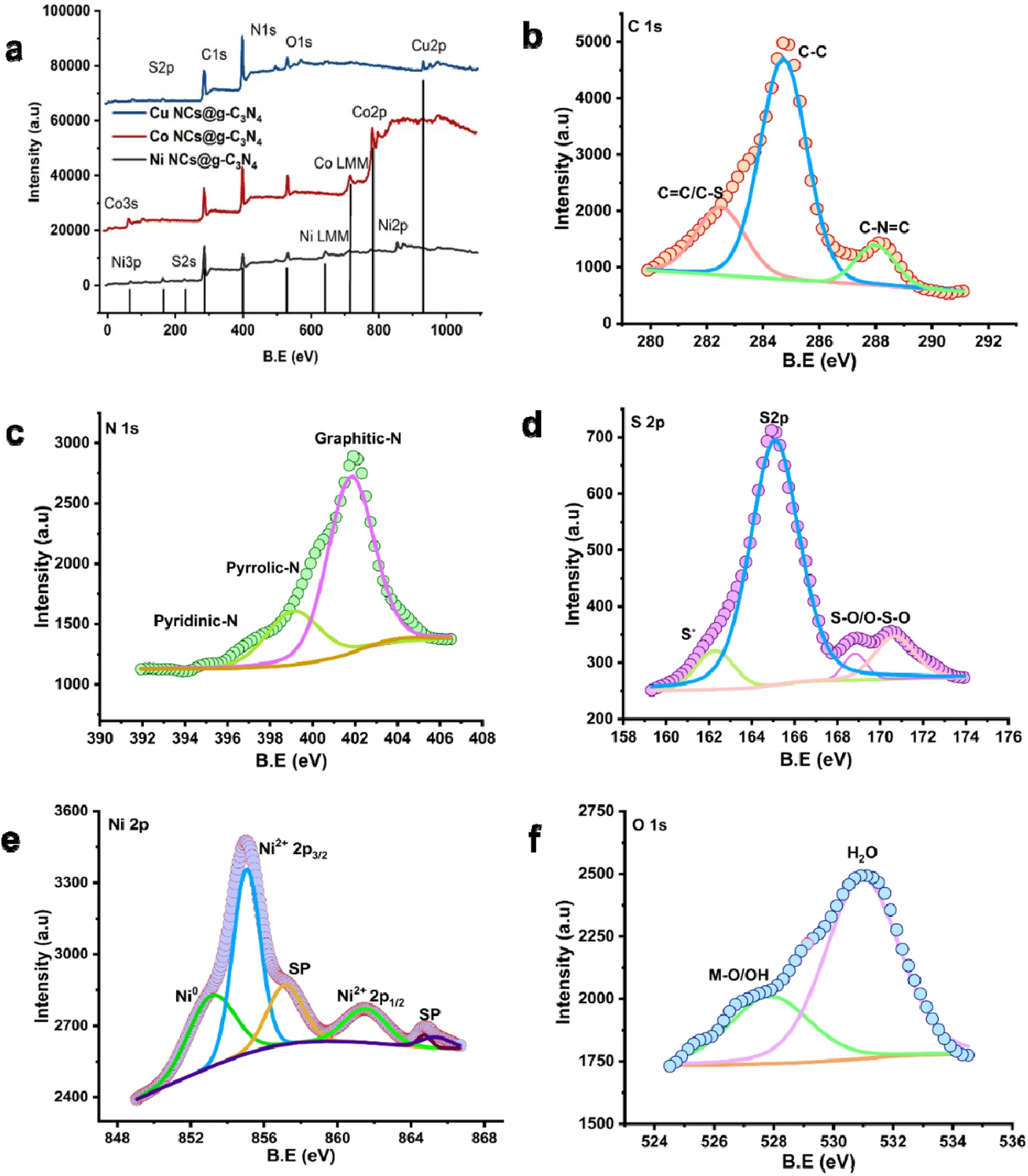
X-ray photoelectron spectroscopy of the thiol-stabilized NCs@NC samples. (a) Survey scan of NiNCs@NC, CoNCs@NC, and CuNCs@NC. (b) Carbon (C 1s), (c) nitrogen (N 1s), (d) sulfur (S 2p), (e) nickel (Ni 2p), and (f) oxygen (O 1s) spectra.

The XPS spectra of CoNCs@NC and CuNCs@NC are more or less similar to those of NiNCs@NC except for the 2p spectra of the metals. The C 1s signal originates from NCs and support, while the presence of the N 1s peak is due to N doping, the S 2p peak represents the presence of thiol, and the O 1s peak is due to atmospheric oxygen or the surface adsorption of OH/H_2_O, as shown in Fig. 6a–d for CuNCs@NC and Fig. S6a–d for CoNCs@NC. The core level spectrum of Cu 2p is shown in Fig. 6e, where the main peaks of Cu 2p_3/2_ and Cu 2p_1/2_ appeared at ∼934.5 eV and ∼953.5 eV, respectively, which are associated with metallic copper (Cu^0^), while the small satellite peaks are the fingerprints of Cu^2+^ that appear from the shake-up process.^62^ The core level spectrum of Co 2p (Fig. 6f) shows the presence of the metal in two different oxidation states (Co^0^ and Co^2+^). The peak towards the lower binding energy is the main peak, appearing at ∼780.2 eV, assigned to Co 2p_3/2_, and the higher binding energy of ∼795.5 eV corresponds to Co 2p_1/2_. These results collectively validate the immobilization of NCs on the NC support.

**Fig. 6:**
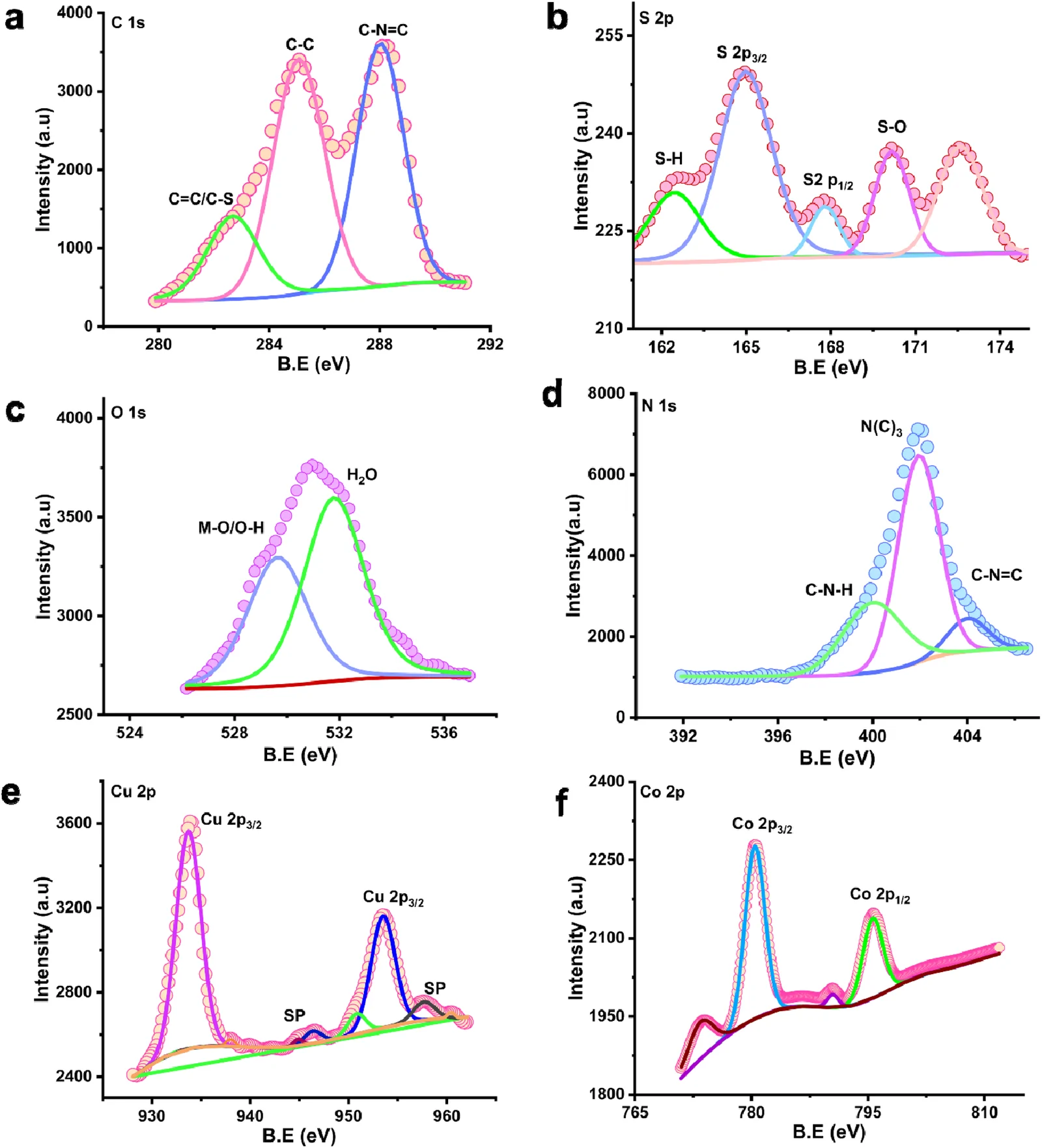
XPS spectra of CuNCs@NC: (a) carbon (C 1s), (b) sulfur (S 2p), (c) oxygen (O 1s), (d) nitrogen (N 1s), (e) copper (Cu 2p) and (f) cobalt (Co 2p) spectra of CoNCs@NC.

A comprehensive structural elucidation obtained through these microscopic and spectroscopic techniques confirms the synthesis of highly pure NCs immobilized on the NC support. HR-TEM and EDX analyses indicate the expected composition of ultrasmall-sized metal aggregates uniformly distributed on the support. FTIR, UV-vis, and PL spectroscopies and XPS analysis validate the metal–ligand and metal–support coordination, electronic contact, and chemical environment. Collectively, all these advanced techniques unequivocally explain and advocate the synthesis of the desired nanoscale materials.

## 4. Electrochemical evaluation

The OER performance of the synthesized materials, NiNCs@NC, CoNCs@NC, and CuNCs@NC, was systematically evaluated using cyclic voltammetry, as shown in Fig. 7a. All three catalysts exhibit the characteristic anodic current associated with oxygen evolution; however, distinct differences in catalytic activity are observed. Among the synthesized materials, NiNCs@NC displays the earliest catalytic onset at 1.45 V *vs.* RHE, while CoNCs@NC and CuNCs@NC initiate oxygen evolution at 1.48 and 1.51 V, respectively. Likewise, NiNCs@NC requires an overpotential of 220 mV@10 mA cm^−2^, which is significantly lower than that of CoNCs@NC (250 mV) and CuNCs@NC (280 mV). Besides the lowest operating potential, NiNCs@NC delivers the highest current density of 171 mA cm^−2^ at 1.60 V, while CoNCs@NC delivers only 120 mA cm^−2^ at 1.62 V and CuNCs@NC produces merely 70 mA cm^−2^ at 1.67 V. These results clearly demonstrate that NiNCs@NC not only initiates oxygen evolution at the lowest potential, but also sustains a substantially higher anodic current, indicating both superior intrinsic catalytic activity and faster oxygen evolution kinetics.

**Fig. 7:**
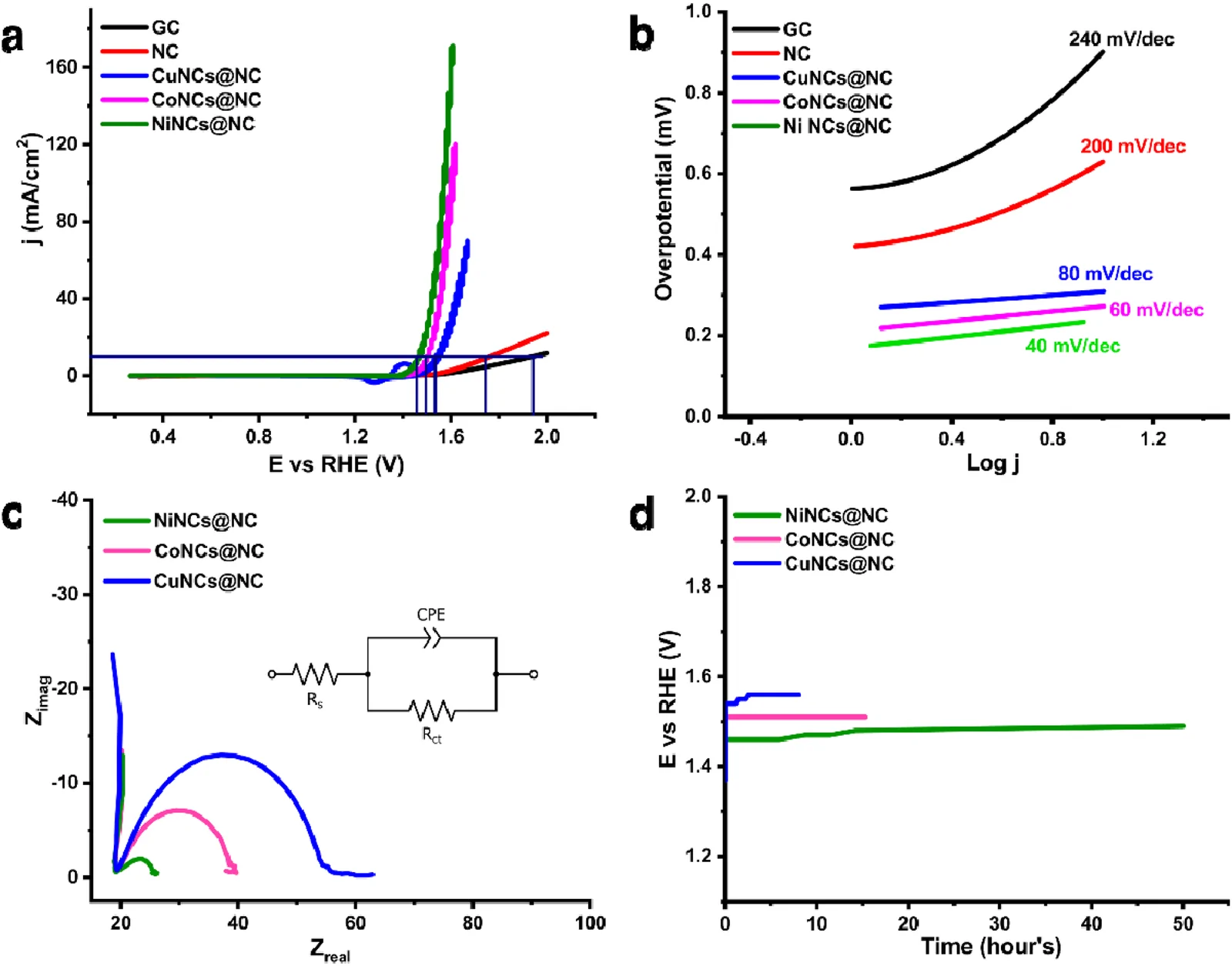
Electrochemical evaluation of the transition metal (Ni, Cu, and Co) nanoclusters (NCs) on N-doped carbon (NC) in comparison with NC and glassy carbon (GC). (a) Cyclic voltammograms of the NCs@NC samples in 1 M KOH. (b) Tafel slopes of the NCs@NC samples. (c) EIS curves of the NCs@NC samples. (d) Chronopotentiometric curves of the NCs@NC samples in 1 M KOH.

The electrochemical performance of the synthesized materials was compared with that of bare NC and GC (Fig. 7a) to understand the synergy between metal NCs and the NC support, and its role in boosting the OER performance. The enhanced OER performance in terms of the onset potential and output current response exemplifies the possible synergistic interactions between NCs and the NC, which simultaneously enhance the intrinsic activity and charge transfer characteristics of all the hybrid materials.

The intrinsic OER kinetics of the synthesized electrocatalysts, NiNCs@NC, CuNCs@NC and CoNCs@NC, were evaluated by Tafel slopes derived from CV polarization curves, as shown in Fig. 7b. It is a prime descriptor of the reaction kinetics, reflecting the rate-determining step of the OER process and the efficiency of charge transfer at the electrode–electrolyte interface. The Tafel slope values of 40 mV dec^−1^, 60 mV dec^−1^ and 80 mV dec^−1^ correspond to NiNCs@NC, CoNCs@NC and CuNCs@NC, respectively. The smallest Tafel slope indicates reasonable reaction kinetics. NiNCs@NC, CoNCs@NC and CuNCs@NC exhibit lower Tafel slopes compared with bare glassy carbon (240 mV dec^−1^) and pristine NC (200 mV dec^−1^), indicating fast electro-catalytic kinetics. The improved performance may have originated from the strong metal–support electronic coupling that facilitates rapid interfacial electron transfer, reduces charge transfer resistance, and optimizes the adsorption–desorption behavior of the oxygenated intermediates, thus being responsible for improving the overall efficiency of the electrode process.^40^

The inherent charge transfer capability of the electrocatalysts was analyzed using Nyquist plots by calculating the charge transfer resistance (*R*_ct_) values (Fig. 7c). The Nyquist plots were nonlinearly fitted using a modified Randle-type equivalent circuit (Fig. 7c inset) consisting of the solution/uncompensated resistance (*R*_s_) in series with a parallel combination *R*_ct_ and a constant phase element (CPE) account for the non-ideal double layer/interfacial capacitance (*R*_s_(*R*_ct_‖CPE)). *R*_ct_ values of 9 Ω, 20 Ω, and 34 Ω are observed for NiNCs@NC, CoNCs@NC, and CuNCs@NC, respectively. The *R*_ct_ trend is well aligned with the kinetic trend derived from the Tafel slopes. This synchronized performance and comparison with bare NC and GC suggest that the internal resistance of the system can be mitigated by the electronic coupling between NCs and the NC support. By comparing the overall performance, it can be stated that the electrocatalytic potential follows the order of NiNCs@NC > CoNCs@NC > CuNCs@NC. Although CoNCs@NC and CuNCs@NC exhibit significantly higher OER activity than bare GC and NC, they remain inferior to that of NiNCs@NC. This implies the inherent potential of the redox couple Ni^2+/3+^ generated in alkaline conditions, a widely accepted active species for the OER.^63^ Furthermore, the role of the support is obvious by virtue of the strong electronic interaction between NiNCs and nitrogen moieties, which is evident from the change in the optical band gap. The lowest optical band gap (2.06 eV) and weakest PL intensity of NiNCs@NC manifest good electrical contact, efficient charge separation, and accelerated electron transfer. Such electronically wired interactions at the electrode–electrolyte interface can judiciously facilitate the OER intermediates and the overall conversion process.

The commercial aspect of these NCs@NC samples was analyzed by performing a long-term stability test. The durability of the electrocatalysts was evaluated using chronopotentiometry at a constant current density of 10 mA cm^−2^ (Fig. 7d). All the NCs@NC samples showed long-term stability for 10 hours without any decline in their performance. However, based on the onset potential and low Tafel slope, it was preferred to extend the stability test of NiNCs@NC. The long-term stability test over an extended period of 50 hours at nearly constant current response under harsh alkaline conditions indicates appreciable performance, better than that of similar materials reported in the literature (Table S1). The long-term stability of the electrocatalysts means that they have an optimized structure, and the effective synergistic interplay between NCs and the NC could dynamically catalyze the process without any loss or leaching of the sample.^64^

Further, the electrochemically active surface area (ECSA) was estimated by cyclic voltammetry (CV), which was performed at different scan rates (10, 20, 30, 40, and 50 mV s^−1^) in the non-faradic potential region (0.0–0.1 V *vs.* Ag/AgCl, equivalent to ∼1.02–1.12 V *vs.* RHE), exhibiting a quasi-rectangular profile, as shown in Fig. S6a–c. As the scan rate increases, the anodic and cathodic current densities increase proportionally while preserving the overall shape of the voltammograms, suggesting rapid ion transport and efficient charge accumulation within the electrochemically accessible surface. The enlargement of the enclosed CV area with increasing scan rate reflects an enhanced capacitive response and good density of accessible active sites. The double-layer capacitance was determined from the linear relationship between the scan rate and capacitive current density. NiNCs@NC exhibited the highest *C*_dl_ value of 374 µF cm^−2^ compared with CuNCs@NC (214 µF cm^−2^) and CoNCs@NC (95 µF cm^−2^), as shown in Fig. S6d. The improved *C*_dl_ and enhanced ECSA may be attributed to the uniform distribution and immobilization of small metal NCs on the conducting support, hindering their agglomeration, promoting the exposure of active catalytic sites, and possibly allowing the penetration of the electrolyte to the metal–support interface. Meanwhile, the surface-coated hydrophobic linkers could facilitate the desorption of molecular oxygen, and thus can regenerate the active sites for the next cycle, as evident from the stability test.

## 5. Conclusions

Inspired by interfacial engineering strategies, this work presents a rational and versatile approach for the immobilization of thiol-stabilized transition metal (Ni, Co, and Cu) nanoscale materials on N-doped carbon. The development of strong electronic contact between small metal aggregates with the support guarantees uniform dispersion, emergence of active sites, and efficient charge transfer, thus enhancing the OER performance. In-depth structural and surface characterization by UV-vis and FTIR spectroscopies, EDX, HR-TEM, and XPS confirmed the successful formation of ultrasmall nanoclusters with homogeneous dispersion, tailored surface coordination, and strong electronic coupling with the conductive carbon framework. Furthermore, PL spectroscopy confirms the most promising interfacial charge transfer for NiNCs@NC. The electrochemical performance was systematically evaluated using CV, EIS, *C*_dl_, Tafel slope, and stability tests, which established a distinct activity trend of NiNCs@NC > CoNCs@NC > CuNCs@NC. Among the synthesized catalysts, NiNCs@NC exhibited the best performance, delivering a low overpotential of 220 mV at 10 mA cm^−2^, with a maximum current density of 171 mA cm^−2^, and excellent long-term stability for >50 hours. The catalytic performance can be attributed to the good electronic contact, synergistic interplay between metals and the support, and abundant exposure of catalytically active sites resulting from the sub-nanometric dimensions. These findings provide a promising foundation for extending this synthetic strategy to heterometallic and high-entropy nanoclusters. Future studies may focus on tailoring the ligand electronic structures to precisely regulate the catalytically active sites. Integrating NCs with advanced conductive architectures, such as MXenes, MOFs, and COFs, could further improve charge transfer and durability.

## Conflicts of interest

The authors declare no conflicts of interest.

## Supplementary Material

RA-OLF-D6RA07054J-s001

## Data Availability

The authors confirm that the data supporting the findings of this study are available within the article and its supplementary information (SI). Furthermore, all related data are available from the corresponding authors on request. Supplementary information is available. See DOI: https://doi.org/10.1039/d6ra07054j.
