# Supplementary material for: Immobilization of transition metal (Ni, Cu, and Co)-based colloidal nanoscale materials for the oxygen evolution reaction
Source: RSC Adv. 2026 Oct 14;16(47):62962–76. doi: 10.1039/d6ra07054j (PMC13667366; doi:10.1039/d6ra07054j)
Supplement: Supplementary file 1 [file RA-016-D6RA07054J-s001.pdf]

Supplementary Information (SI) for RSC Advances.
This journal is © The Royal Society of Chemistry 2026
Supplementary Information (SI) for RSC Advances.
This journal is © The Royal Society of Chemistry

Supplementary Information

Immobilization of Transition Metals (Ni, Cu, and Co) Colloidal Nanoscale Materials for the Oxygen Evolution Reaction

Sajid Ullah[a], Syeda Tabeer Zahra[a], Khizer Hayat Khan[a], R. Nawaz[b], Norah Alomayrah[c], M. S. Al-Buriahi[d], Aiyeshah Alhodaib[e], Zeinebou Yahya[f], Ziaur Rehman[a*], Akhtar Munir[a*]

[a]Department of Chemistry, Quaid-i-Azam University, 45320, Islamabad, Pakistan

[b]Center for Applied Mathematics and Bioinformatics (CAMB), Gulf University for Science and Technology, 32093 Hawally, Kuwait

[c]Department of Physics, College of Science, Princess Nourah bint Abdulrahman University, P.O. Box 84428, Riyadh 11671, Saudi Arabia

[d]Department of Physics, Sakarya University, Sakarya, Turkey

[e]Department of Physics, College of Science, Qassim University, Buraydah 51452, Qassim, Saudi Arabia

[f]Department of Physics, College of Science, Qassim University, 51452, Buraidah, Al-Qassim, Saudi Arabia

** Corresponding Author*

*Email: akhtarmunir@qau.edu.pk (A. Munir), zrehman@qau.edu.pk (**Z. Rehman**)*

Optical Band Gap Calculation

The optical band gap energy (Eg) of the catalysts was calculated using the Tauc-Davis-Mott (TDM) model based on the UV-Vis absorption spectra.[1] According to the TDM relation

$$\alpha h\nu = B^2 (h\nu - E_g)^n \qquad \textbf{(Eq. 1)}$$

In Eq.1, **α** is the optical absorption coefficient, **h** is Planck's constant, **ν** is the frequency of the incident photon, **B** is the Tauc band-tailing parameter, e.g. a material-specific constant associated with the transition probability, E_g is the optical band gap energy, and n is the transition index, which depends on the nature of the electronic transition. The values of n are 1/2, 2, 1/3, 3 (direct allowed, indirect allowed, direct forbidden, and indirect forbidden transitions, respectively). For the calculation of band gap energy, the indirect allowed transition (n = 2) was employed, resulting in the most acceptable description of the adsorption edge, yielding the best fit for Tauc analysis.

For indirect transition n = 2

$$\alpha h\nu = B^2 (h\nu - E_g)^2$$

By taking the square root on both sides, we get,

$$\alpha h\nu^{1/2} = B (h\nu - E_g)$$

The optical absorption coefficient (**α**) is derived from the experimentally measured absorbance **(A)** and sample cuvette thickness **(t)** via the Beer-Lambert relation.

$$\alpha = \frac{2.303\,A}{t}$$

The photon energy **(hν)** was obtained from the corresponding wavelength using

$$h\nu = \frac{hc}{\lambda}$$

The optical band gap energy was determined from the Tauc plot by linear fitting of the absorption edge region followed by the extrapolation to $(\alpha h\nu)^{1/2} = 0$. The intercept of the fitted line on the photon energy axis **(hν)** yields the band gap value.[2]

$$(\alpha h\nu)^{1/2} = B\,(h\nu - E_g) \qquad \text{(straight line equation)}$$

$$(\alpha h\nu)^{1/2} = B\,(h\nu - E_g)$$

$$y \quad = \; mx + c$$

y = $(\alpha h\nu)^{1/2}$ (dependent variable)

x= $h\nu$ (eV) (independent variable)

m= B (slope)

c= -BE_g (y-intercept)

At x intercept, y=0

$$0 = B.h\nu - B.E_g \qquad [\text{since } (\alpha h\nu)^{1/2} = 0]$$

$$B.\ h\nu = B.E_g$$

$$E_g = h\nu$$

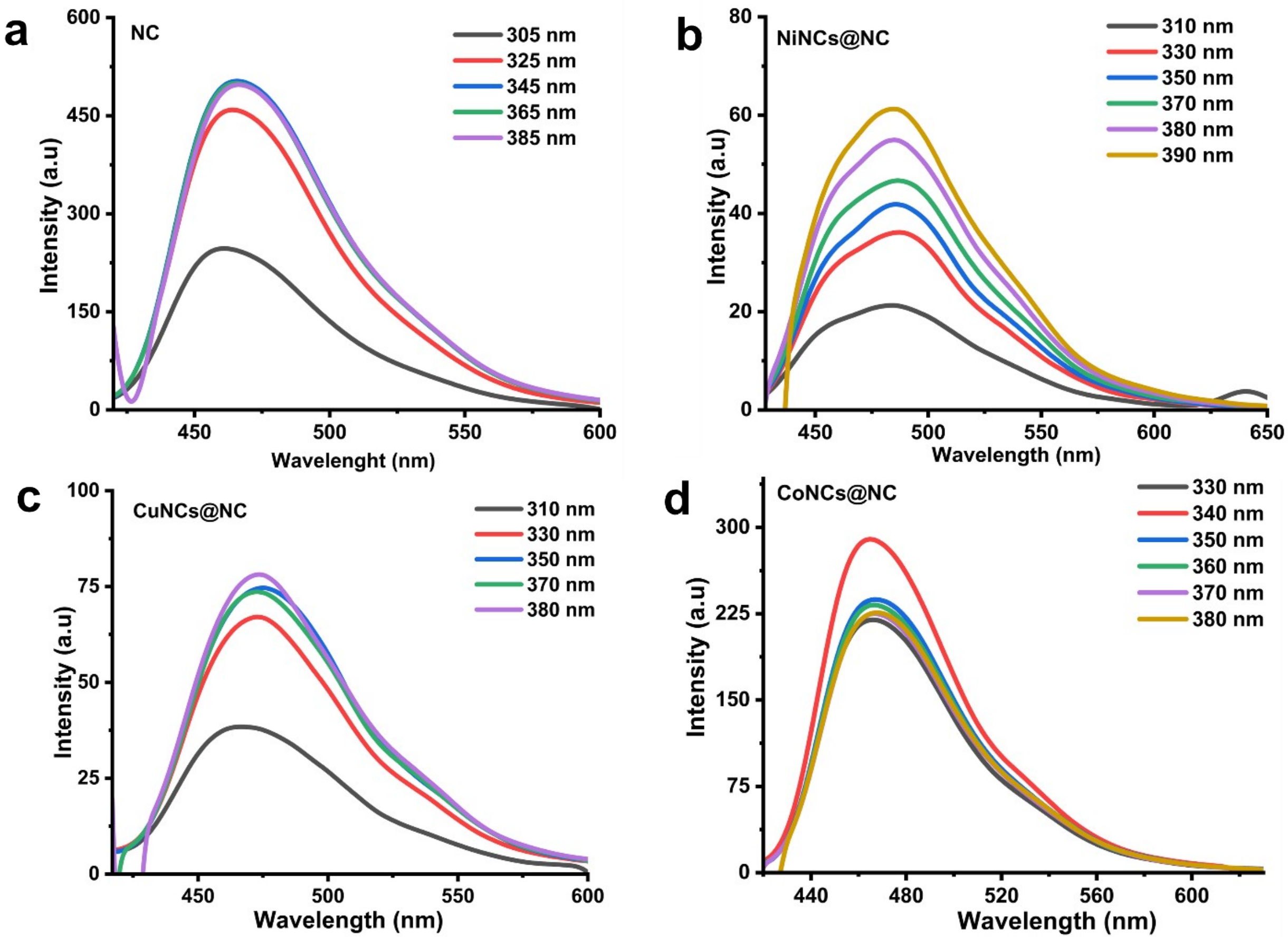

Figure S1. (a) PL emission spectra of NC recorded at excitation wave lenth 305-385nm. Corresponding PL emission spectra of (b)NiNCs@NC**,** (c) CoNCs@NC, and (d)CuNCs@NC measured at various excitation wavelengths.

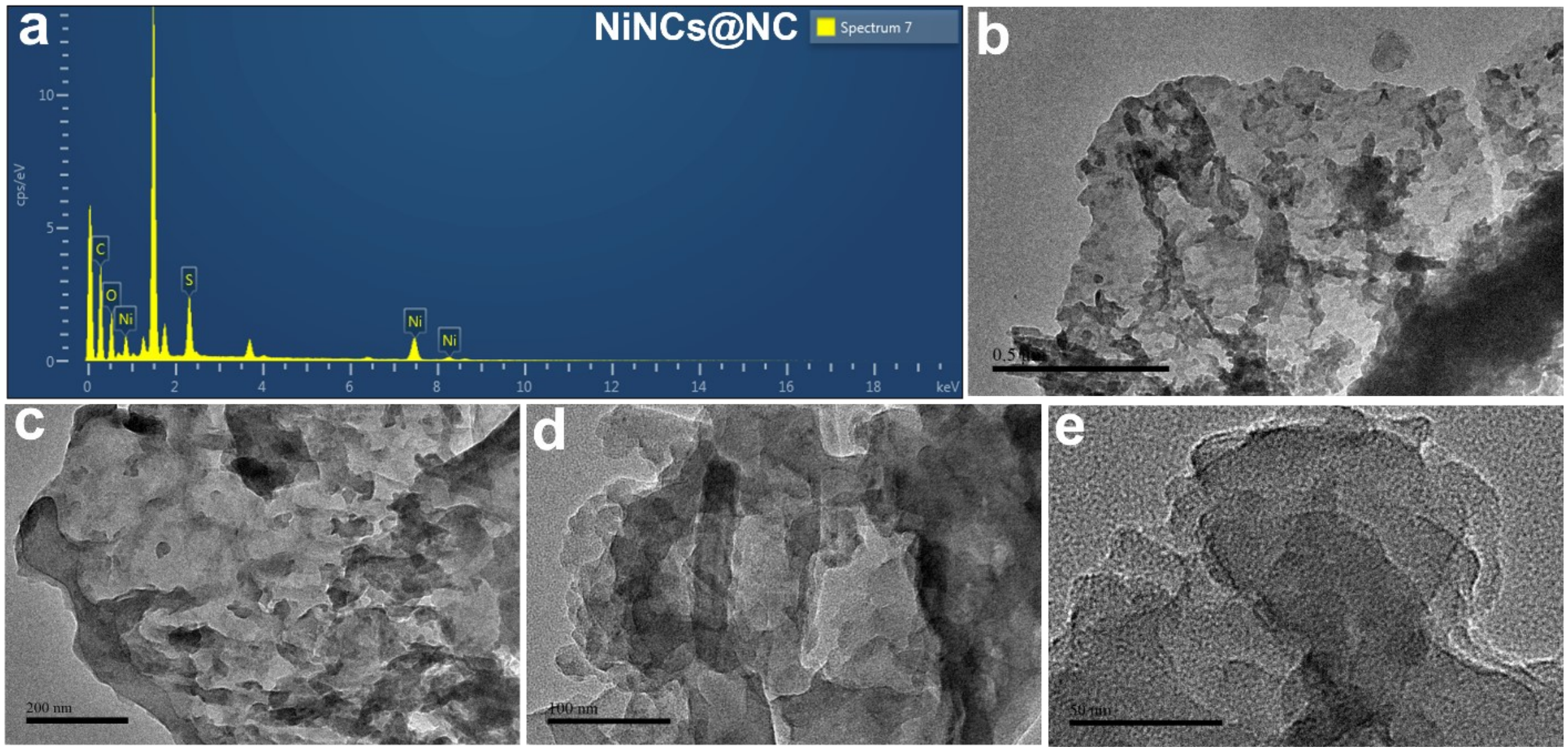

Figure S2. (a) EDX spectra of NiNCs@NC and HRTEM images of NiNCs@NC: (b) 0.5 µm (c) 200 nm (d) 100 nm (e) 50 nm

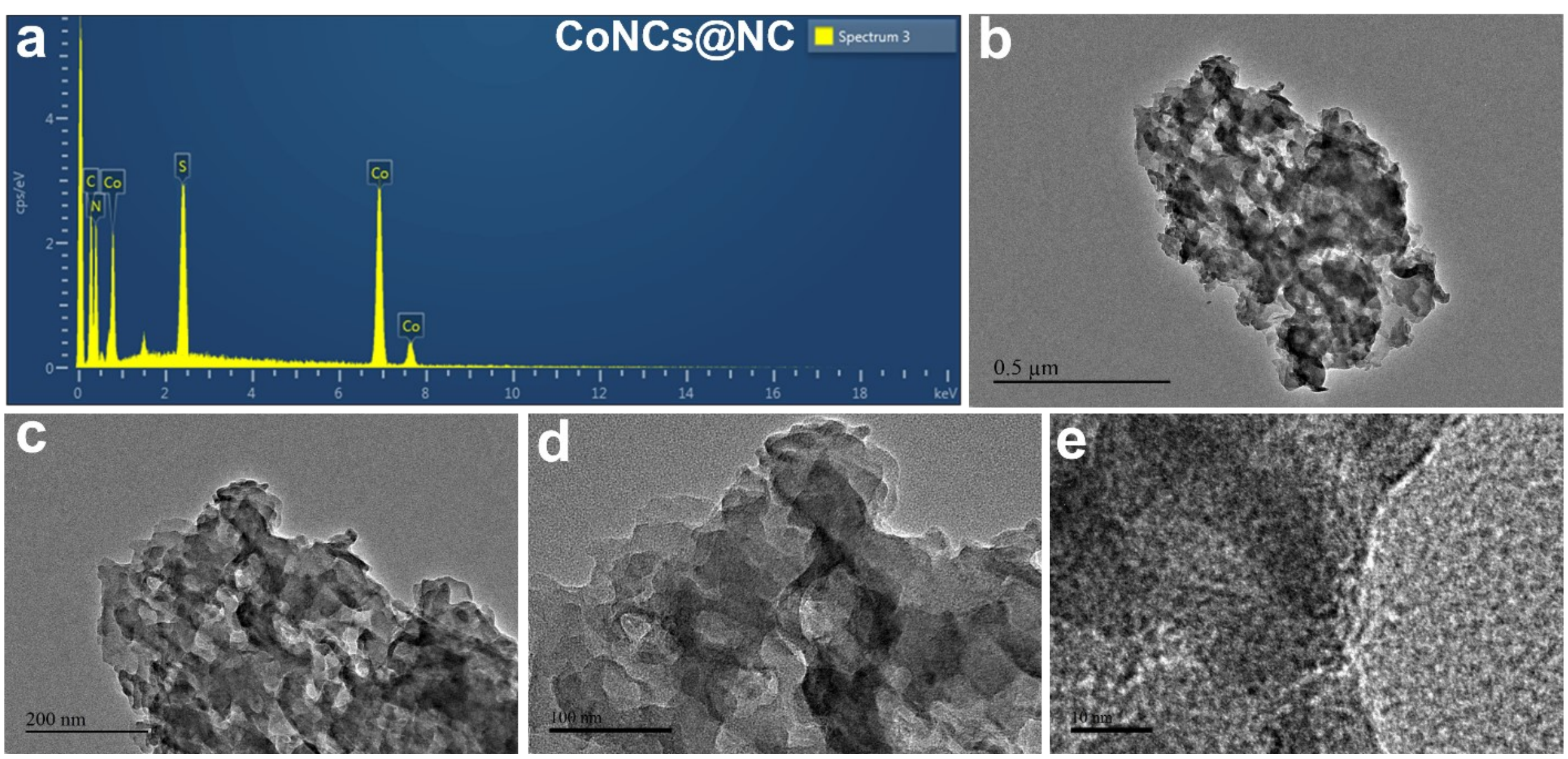

Figure S3. (a) EDX spectra of CoNCs@NC and HRTEM images of CoNCs@NC (b) 0.5 µm (c) 200 nm (d) 100 nm (e) 10 nm

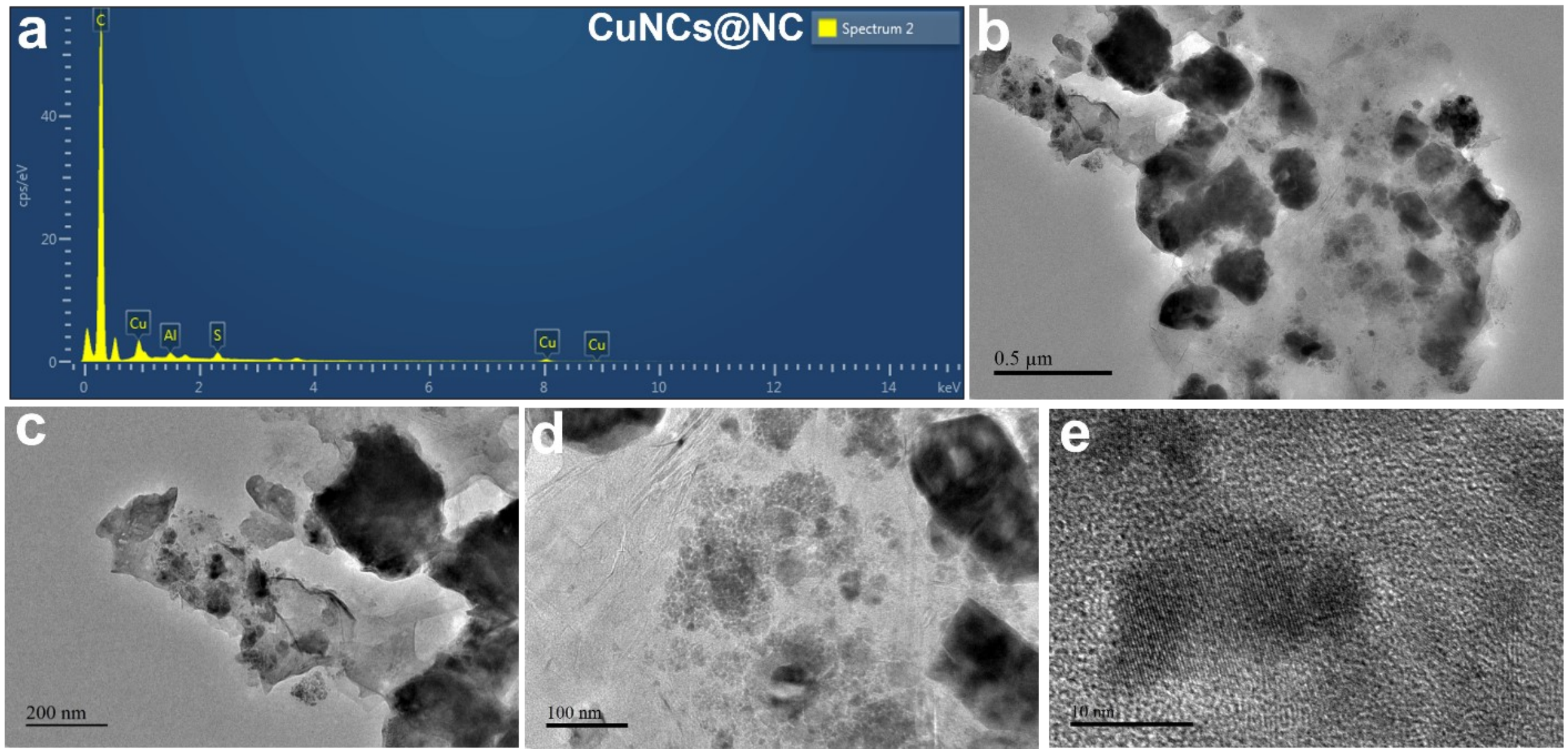

Figure S4. (a) EDX spectra of CuNCs@NC and HRTEM images of CuNCs@NC: (b) 0.5 µm, (c) 200 nm, (d) 100 nm, (e) 10 nm

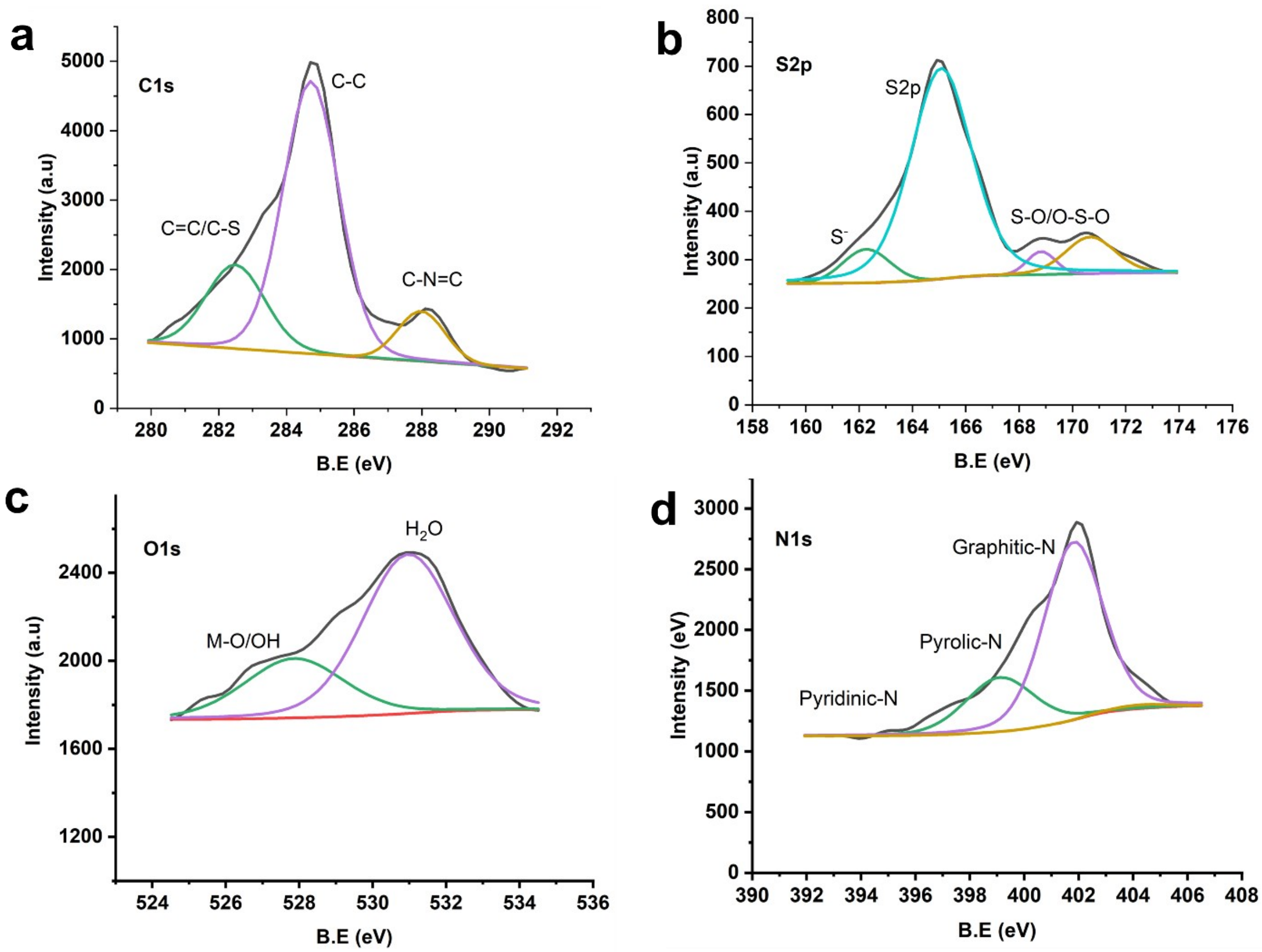

Figure S5. XPS spectra of CoNCs@NC (a) Carbon (C1s), (b) Nitrogen (N 1s), (c) Sulfur (S2p), and (d) Oxygen (O1s).

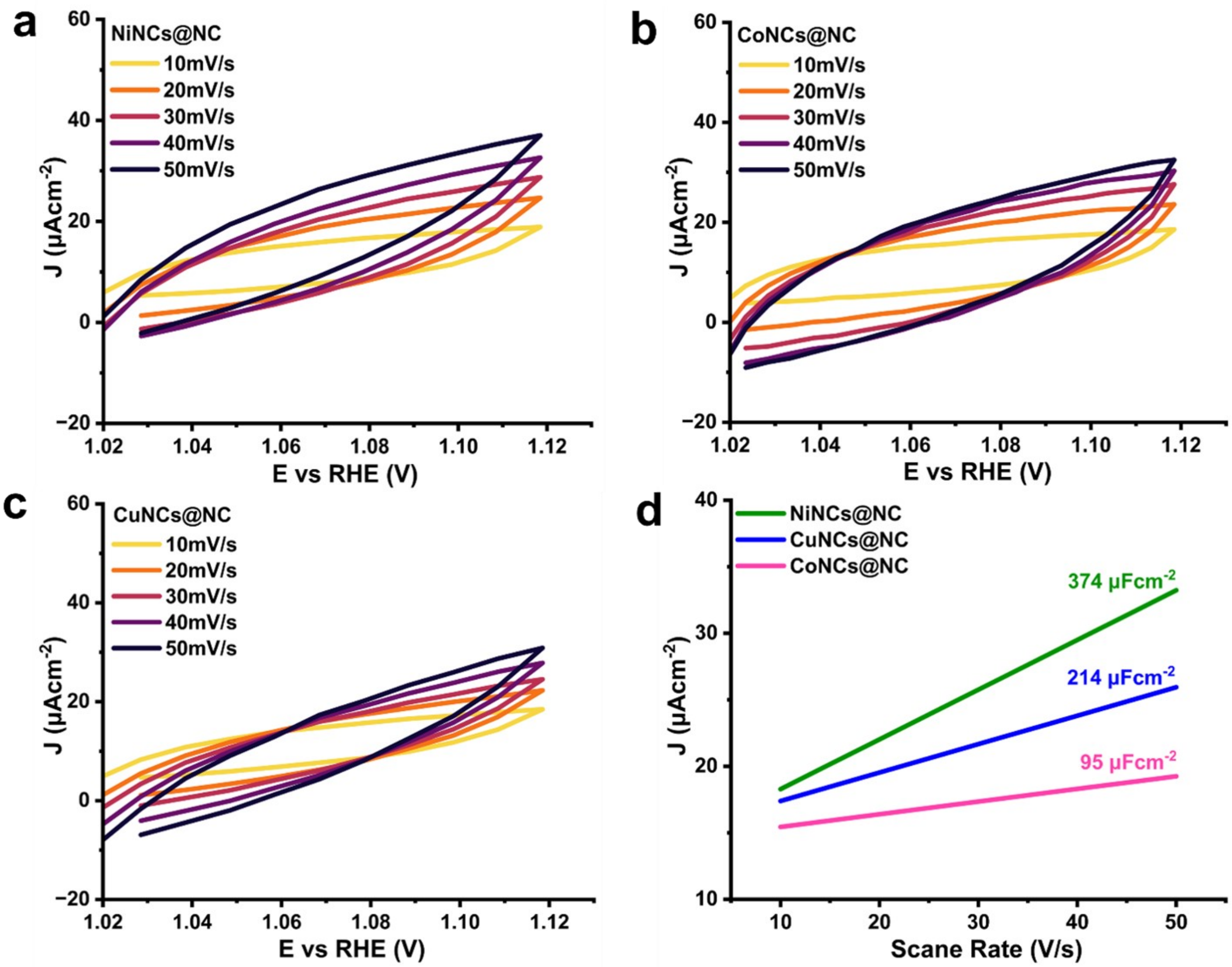

Figure S6. Cyclic voltammograms of (a) NiNCs@NC, (b) CoNCs@NC, (c) CuNCs@NC recorded at different scan rate in the non-faradaic potential region in 1 M KOH, and (d) Linear fit of capacitive current density vs scan rate and the slope represent $C_{dl,}$ which is proportional to ECSA.

Catalyst Ink Preparation and Mass Loading

For electrochemical measurements, 5 mg of the synthesized supported metal nanoclusters (NiNCs@NC, CoNCs@NC, and CuNCs@NC) were dispersed in 400 µL of isopropanol (2-propanol), followed by the addition of 10 µL of 5% Nafion solution as a binder. The resulting catalyst ink was ultrasonicated to obtain a homogeneous suspension and to minimize particle agglomeration. Prior to catalyst deposition, the glassy carbon electrode (GCE, 3mm diameter, geometric area = 0.07 cm^{-2}) was mechanically polished using alumina slurry on a micro-cloth polishing pad, thoroughly rinsed with deionized water, and dried under ambient conditions. Subsequently, 10 µL of the catalyst ink was drop-cast onto the clean GCE surface and allowed to dry at room temperature for sufficient time, forming a uniform catalytic film. Based on the ink composition, the catalyst loading on the electrode was approximately 0.122 mg (122 µg), corresponding to a mass loading of 1.74g cm^{-1}. To ensure experimental reproducibility, all working electrodes were prepared in duplicate, and the consistency of the catalyst loading was verified gravimetrically using a microbalance. All the electrochemical measurements were performed in 1 M KOH aqueous electrolyte at room temperature.

Theoretical Catalyst Loading Mass on GC Electrode

Catalyst Mass in grams = 5 mg

Dispersion Solvent = Isopropanol = 400 µL + Nafion = 10 µL = 410 µL

Catalyst Concentration = $\frac{5\ mg}{410 \mu L}$ = 0.0122 mg μL^{-1}

Catalyst deposited in 10 µL = 0.0122 mg μL^{-1} = 122 µg

Mass Loading = $\frac{0.0122\ mg}{0.07\ cm^2}$ = **1.74 $mgcm^{-2}$**

Actual catalyst loading Mass on GC Electrode

Table S1. Comparison of OER activity of various catalyst with this work

Electrocatalyst	Overpotential @ 10mAcm^{-2}	Tafel Slope (mV/dec)	Stability (hours)	Reference
NiNCs@NC	1.45 (onset)	40	50	**This work**

Actual Loading = Weight of electrode after deposition - Weight of electrode before deposition

$$L = 4.6012\ g - 4.6000\ g$$

$$L = 0.00012\ g = \mathbf{0.12\ mg}$$

As the GCE surface area is 0.07 cm^2, the catalyst mass loading is **1.71 mg cm^{-2}**.

Actual and theoretical calculations for the mass loading of catalyst on GCE are almost the same.

Electrochemical active surface area (EASA)

Double layer charging current is directly proportional to scan rate and double layer capacitance.

$$(i_c) = v\ C_{dl}$$

Where 'v' is the scan rate and C_{dl} is the double-layer capacitance, equal to the slope of the plot of 'v' *vs* capacitive current density.

$$EASA = C_{DL}/C_s$$

where Cs is the specific capacitance of a sample smooth surface under the specific condition of electrolyte and C_{DL} is the double layer capacitance in the non-faradaic potential region of voltammogram. The reported value of *Cs* varies from 0.033 mF to 0.056 mF in alkaline conditions.[3] Hence, we chose an average value (0.040 mF = 40μF) to get the average value of EASA of our catalyst.

NiNCs@NC = 374 μFcm^2 / 40μF 9.35 cm^2

CuNCs@NC = 214 μFcm^2 / 40μF 5.35 cm^2

CoNCs@NC = 95 μFcm^2 / 40μF 2.40 cm^2

	220 mV			
CoNCs@NC	1.48 (onset)	60	15	**This work**
	250 mV			
CuNCs@NC	1.51 (onset)	80	10	**This work**
	280 mV			
5L-CoOx	263	68.7	72	[4]
FeN_4–Ru_6–NiN_4@PCA	350	80	12	[5]
MOF-FE-NCs@NiO-NS	280	84	250	[6]
Li_x RuO_2	156	83.3	70	[7]
Se-FeNi-LDH	240	37	120	[8]
Fe/Ce–Ni_2P	250	38	100	[9]
Ni-MoO_3	340	78.4	24	[10]
$NiFeCoCrW_{0.2}$	220	36.8	90 days	[11]
$Ni_x(FeCoCrCu)_{100-x}$	292	88.4	100	[12]
Cu_8 embedded in $[\alpha\text{-}SiW_{11}O_{39}]^{8-}$	580	62	10	[13]
CoPt-R10	282	93.7	50	[14]
NiSe NW	200	58	10	[15]
Co-N/GF	313	84	30	[16]
Ni NPs-N doped graphene	280	45	10	[17]
NiO–Ni/NF	293	101.1	20	[18]
NiCO@NC (N-doped carbon nanofiber)	530	98	7	[19]
NiFe LDH	229	62.9	2000 cycles	[20]
NiO-WS_2	190	83.1	40	[21]

References

1. S. G. Prasad, C. Lal, K. R. Sahu, A. Saha and U. De, Spectroscopic Investigation of Degradation Reaction Mechanism in γ-Rays Irradiation of HDPE, *Biointerface Res. Appl. Chem.*, 2020, **11**, 9405-9419.
2. P. Negro, F. Cesano, S. Casassa and D. Scarano, Combined DFT-D3 Computational and Experimental Studies on g-C_3N_4: New Insight into Structure, Optical, and Vibrational Properties, *Materials*, 2023, **16**, 3644.
3. A. Munir, T. u. Haq, A. Qurashi, H. U. Rehman, A. Ul-Hamid and I. Hussain, Ultrasmall Ni/NiO nanoclusters on thiol-functionalized and-exfoliated graphene oxide nanosheets for durable oxygen evolution reaction, *ACS Appl. Energy Mater.*, 2018, **2**, 363-371.
4. S. Kwon, B. Wang, J. Y. Heo, M. K. Kim, B. Song, H. B. Bae, J. H. Lee and H. M. Jeong, The role of chemical bond contraction induced via nanoclusterization of cobalt oxide triggering robust lattice oxygen mechanism during the oxygen evolution reaction, *Appl. Catal. B: Environ.*, 2026, **394**, 126857.
5. Y. Zhang, K. Kong, H. Jie, X. Jin, L. Tian, Y. Liu, Z. Pan, S.-J. Hwang, L. Li and Z. Wang, Engineering Fe–Ni Dual-Atom Sites Via Ru Nanoclusters on 3D Carbon Aerogel for Enhanced Bifunctional Oxygen Electrocatalysis, *Nano-Micro Lett.*, 2026, **18**, 404.
6. R. Gunaseelan, S. Marimuthu and G. Maduraiveeran, Nanoclusters of Metal–Organic Frameworks on NiO Nanosheets for Water Splitting, *ACS Appl. Nano Mater.*, 2026, **9**, 661-674.
7. X. Tan, S. Wang, C. Zhang, Y. deng, Y. Lian and J. Bai, Towards boosting activity and stability of RuO_2-based electrocatalysts for acidic oxygen evolution reaction, *J. Alloys Compd*, 2026, **1065**, 188104.
8. A. Hassan, G. E. Khedr, A. K. Gomaa, L. H. Abdel-Rahman, H. M. El-Sagher and N. K. Allam, Selenium-activated monolithic FeNi layered double hydroxide electrodes: binder-free, self-supported architectures for durable alkaline oxygen evolution, *RSC Adv.*, 2026, **16**, 22269-22281.
9. L. Zhao, L. Wang, R. Shi, H. Xu, Z. Zeng, X. Lang, Y. Huang, Y. Liu, X. Liao and M. Nie, Fe/Ce codoped Ni_2P catalyst to enhance alkaline oxygen evolution reaction, *ACS Appl. Energy Mater.*, 2025, **8**, 5474-5481.
10. A. K. Verma, S. Atif, A. Padhy, T. S. Choksi, P. Barpanda and A. Govind Rajan, Robust oxygen evolution on Ni-doped MoO_3: overcoming activity–stability trade-off in alkaline water splitting, *Chem & Bio Eng.*, 2025, **2**, 241-252.

11. T. Zhang, H.-F. Zhao, Z.-J. Chen, Q. Yang, N. Gao, L. Li, N. Luo, J. Zheng, S.-D. Bao and J. Peng, High-entropy alloy enables multi-path electron synergism and lattice oxygen activation for enhanced oxygen evolution activity, *Nat. Commun.*, 2025, **16**, 3327.

12. Y. Pan, F. Zhao, G. Ji, S. Xu, R. Li, H. Zhou, R. Zhao, D. Zhao and F. Wu, Electrocatalytic performance of Ni-based high-entropy alloys with unique structural features in alkaline and seawater electrolysis under−10° C, *Int. J. Hydrogen Energy*, 2025, **127**, 147-159.

13. J. Lin, X. Chen, N. Wang, S. Liu, Y. Chen, Z. Tian and Z. Ruan, Octanuclear Cu(II) cluster–tungstosilicate composite as efficient electrocatalyst for oxygen evolution reaction at near-neutral pH, *Sustain. Energy Fuels*, 2022, **6**, 223-230.

14. Y. Yan, Z.-A. Ye, Y. Xu, J. Jiang, R. Zhao, F. Guo, C. Li and M. Chen, Ultralow platinum-doped self-supported CoPt alloy nanosheets for efficient overall water splitting, *Chem. Commun.*, 2025, **61**, 9944–9947.

15. Y. Li, D. Yan, Y. Zou, C. Xie, Y. Wang, Y. Zhang and S. Wang, Rapidly engineering the electronic properties and morphological structure of NiSe nanowires for the oxygen evolution reaction, *J. Mater. Chem. A*, 2017, **5**, 25494-25500.

16. Y. Tong, X. Yu, H. Wang, B. Yao, C. Li and G. Shi, Trace Level Co–N Doped Graphite Foams as High-Performance Self-Standing Electrocatalytic Electrodes for Hydrogen and Oxygen Evolution, *ACS Catal.*, 2018, **8**, 4637-4644.

17. Y. Xu, W. Tu, B. Zhang, S. Yin, Y. Huang, M. Kraft and R. Xu, Nickel Nanoparticles Encapsulated in Few-Layer Nitrogen-Doped Graphene Derived from Metal–Organic Frameworks as Efficient Bifunctional Electrocatalysts for Overall Water Splitting, *Adv. Mater*, 2017, **29**, 1605957.

18. Z. Yue, W. Zhu, Y. Li, Z. Wei, N. Hu, Y. Suo and J. Wang, Surface Engineering of a Nickel Oxide–Nickel Hybrid Nanoarray as a Versatile Catalyst for Both Superior Water and Urea Oxidation, *Inorg. chem.*, 2018, **57**, 4693-4698.

19. Y. Fu, H. Y. Yu, C. Jiang, T. H. Zhang, R. Zhan, X. Li, J. F. Li, J. H. Tian and R. Yang, NiCo Alloy Nanoparticles Decorated on N-Doped Carbon Nanofibers as Highly Active and Durable Oxygen Electrocatalyst, *Adv. Funct. Mater.*, 2018, **28**, 1705094.

20. Y. Wang, M. Qiao, Y. Li and S. Wang, Tuning Surface Electronic Configuration of NiFe LDHs Nanosheets by Introducing Cation Vacancies (Fe or Ni) as Highly Efficient Electrocatalysts for Oxygen Evolution Reaction, *Small*, 2018, **14**, 1800136.

21. D. Wang, Q. Li, C. Han, Z. Xing and X. Yang, When NiO@Ni Meets WS_2 Nanosheet Array: A Highly Efficient and Ultrastable Electrocatalyst for Overall Water Splitting, *ACS Cent. Sci.*, 2017, **4**, 112-119.
